# Light-enhanced VEGF_121_/rGel induce immunogenic cell death and increase the antitumor activity of αCTLA4 treatment

**DOI:** 10.3389/fimmu.2023.1278000

**Published:** 2023-12-19

**Authors:** Ane Sager Longva, Kristian Berg, Anette Weyergang

**Affiliations:** Department of Radiation Biology, Institute for Cancer Research, Norwegian Radium Hospital, Oslo University Hospital, Oslo, Norway

**Keywords:** immune check point inhibitor (ICI), photodynamic therapy, photochemical internalization (PCI), vascular targeting, immunogenic cell death (ICD), targeted toxin, vascular endothelial growth factor

## Abstract

**Background:**

Immune-checkpoint inhibitors (ICIs) represent a revolution in cancer therapy and are currently implemented as standard therapy within several cancer indications. Nevertheless, the treatment is only effective in a subset of patients, and immune-related adverse effects complicate the improved survival. Adjuvant treatments that can improve the efficacy of ICIs are highly warranted, not only to increase the response rate, but also to reduce the therapeutic ICI dosage. Several treatment modalities have been suggested as ICI adjuvants including vascular targeted treatments and photodynamic therapy (PDT). Photochemical internalization (PCI) is a drug delivery system, based on PDT. PCI is long known to generate an immune response in murine models and was recently shown to enhance the cellular immune response of a vaccine in a clinical study. In the present work we evaluated PCI in combination with the vascular targeting toxin VEGF_121_/rGel with respect to induction of immune-mediated cell death as well as *in vitro* ICI enhancement.

**Methods:**

DAMP signaling post VEGF_121_/rGel-PCI was assessed in CT26 and MC38 murine colon cancer cell lines. Hypericin-PDT, previously indicated as an highly efficient DAMP inducer (but difficult to utilize clinically), was used as a control. ATP release was detected by a bioluminescent kit while HMGB1 and HSP90 relocalization and secretion was detected by fluorescence microscopy and western blotting. VEGF_121_/rGel-PCI was further investigated as an αCTLA enhancer in CT26 and MC38 tumors by measurement of tumor growth delay. CD8+ Dependent efficacy was evaluated *in vivo* using a CD8+ antibody.

**Results:**

VEGF_121_/rGel-PCI was shown to induce increased DAMP signaling as compared to PDT and VEGF_121_/rGel alone and the magnitude was found similar to that induced by Hypericin-PDT. Furthermore, a significant CD8+ dependent enhanced αCTLA-4 treatment effect was observed when VEGF_121_/rGel-PCI was used as an adjuvant in both tumor models.

**Conclusions:**

VEGF_121_/rGel-PCI describes a novel concept for ICI enhancement which induces a rapid CD8+ dependent tumor eradication in both CT26 and MC38 tumors. The concept is based on the combination of intracellular ROS generation and vascular targeting using a plant derived toxin and will be developed towards clinical utilization.

## Background

The overall goal for cancer immunotherapy is to harness the immune system to recognize and kill cancer cells. The greatest progress in cancer immunotherapy has been within the field of immune checkpoint inhibitors (ICIs) which function by suppressing immune-inhibitory signaling and thereby increase the activity of activated cytotoxic T-cells (CD8+). Currently, clinically relevant ICIs act on the CTLA-4 or PD-1/PD-L1 checkpoints with main function in the priming- and effector-phase of the immunoresponse (1). The CTLA-4 checkpoint works as an inhibitory signal for T-cell activation by antigen presenting cells, and αCTLA4 antibodies release this suppression causing an increase in CD8+ T-cells (1). The PD-1/PD-L1 checkpoint works as an inhibitory signal when a CD8+ cell recognize a tumor cell, and blocking this signal with αPD-1 or αPD-L1 antibodies increases CD8+ mediated tumor cell death (1). Clinical efficacy of ICIs has been demonstrated in several cancer types including melanoma, renal-, colorectal- and non-small-cell lung-cancer and clinical approval is currently obtained for 6 drugs in this class of therapeutics. However, ICIs are only effective in a subset of patients and clinical benefit is estimated up to only 30-40%, and often less (1, 2). Furthermore, immune related adverse effects (irAEs) represent a considerable limitation for ICI therapy (3). Grade ≥ 3 toxicity is reported in 37% of patients receiving ipilimumab at 3mg/kg and 58% at 10mg/kg (4). Altogether, adjuvant therapy that enhances the efficacy of ICI without increasing toxicity is highly warranted. Several studies have indicated ICIs as most effective in patients with high mutational tumor burden (MTB) as well as active antitumor immune-responses prior to therapy (e.g increased accumulation of T-lymphocytes within the lesion) (5). Adjuvants which increase antitumor immunity are therefore expected as ICI-enhancers. Furthermore, there is a general assumption that vascular targeting, using angiogenic inhibitors, can increase the effect of ICIs (6–8). Thus, treatment modalities which target tumor vasculature and increase antitumor immunity should hold promise as ICI adjuvants. VEGF_121_/rGel is a vascular targeting toxin consisting of VEGF_121_ fused to the type 1 ribosome inactivating protein toxin gelonin (9, 10). We have previously shown that the anticancer efficacy of VEGF_121_/rGel can be increased by the intracellular delivery technology photochemical internalization (PCI) (11, 12), and that VEGF_121_/rGel-PCI induce a strong immune-mediated effect (11). Thus, VEGF_121_/rGel-PCI may function as an ICI enhancer.

Photochemical internalization (PCI) is a clinical relevant technology which uses light activation of amphiphilic photosensitizers to stimulate intracellular drug delivery by rupturing endocytic vesicles loaded with anticancer drugs (13–15). PCI is based on the principles of photodynamic therapy (PDT) where light activation of photosensitizers is used for the treatment of cancer as well as other diseases (16). PCI may therefore be recognized as a combination of PDT (with an amphiphilic photosensitizer) and the drug of interest. PDT induces considerable inflammation in the treated area and also induces immunogenic cell death (ICD) including the release of damage-associated molecular patterns (DAMPs) (17). These molecules act as danger signals and stimulate recruitment and maturation of antigen-presenting cells (APCs). Excretion of heat shock proteins (HSPs), high-mobility group box1 (HMGB1), ATP and calreticulin (CRT) are recognized DAMP signals in PDT-induced ICD. PDT with several photosensitizers has been shown to enhance the effect of ICI, and the ICD as well as treatment induced inflammation has been argued as the mechanisms behind.

**Figure 1:**
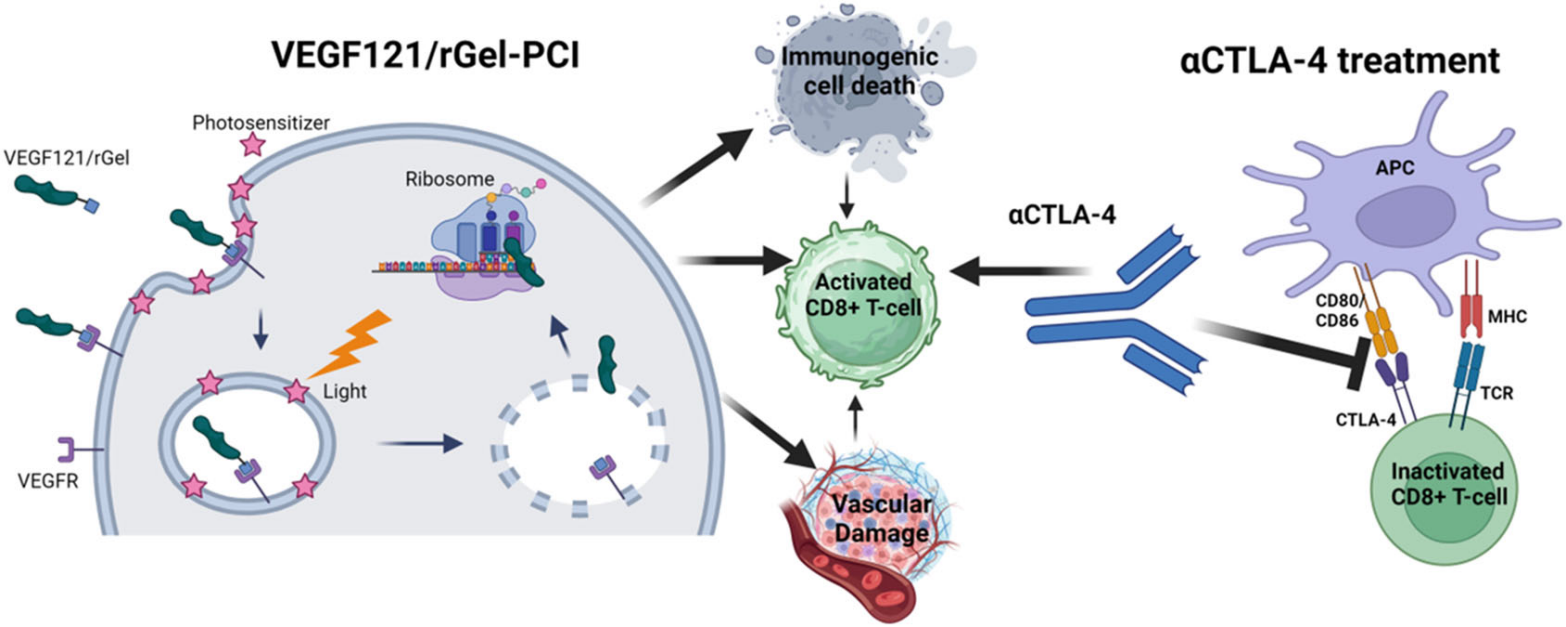
Schematic illustration of mechanisms induced by VEGF121/rGel-PCI in combination with αCTLA4 treatment Created with BioRender.com.

Previous studies has pointed towards VEGF121/rGel as an inducer of vascular damage and immunogenic cell death as well as an activator of CD8+ T-cells (**Figure 1**). All of these mechanisms points towards VEGF121/rGel-PCI as an enhancer of ICI. In the present study VEGF_121_/rGel-PCI was investigated as an adjuvant to a murine αCTLA-4 antibody in two murine colon cancer allografts using (**Figure 1**). A significant CD8+ dependent enhanced αCTLA4 treatment effect was observed when VEGF_121_/rGel-PCI was used as an adjuvant. The effect of the combination was most pronounced in the CT26 model the first days after treatment resulting in 5/6 animals in complete remission (CR) at the highest αCTLA4 dose compared to no CRs in any of the control groups. VEGF_121_/rGel-PCI enhanced αCTLA4 treatment was also significantly increased as compared to using PDT as an enhancer. Studying *in vitro* DAMP signaling following VEGF_121_/rGel-PCI in both models reviled an increased amount of HSP90, HMGB1 and ATP secretion as compared to PDT using both TPCS_2a_ and Hypericin as photosensitizers, indicating that gelonin inhibition of protein synthesis induces ICD signaling.

## Methods

### Cell lines and animal models

CT26.WT (CRL-2638) (ATCC, Manassas, VA, USA) and MC-38 (BE12-604F/U1) (Kerafast, Boston, MA,USA) were maintained according to the providers. Balb/cJRj and C57BL/6 JRj were obtained from Janiver labs, Saint Berthevin, Pys-de-la-Loire, France and bred at our institution. For more information see **Supplementary Material**.

### Drugs, chemicals and light source

Meso-tetraphenyl chlorin disulphonate (TPCS_2a_) was obtained from PCI Biotech AS (Oslo Norway) VEGF_121_/rGel, from now indicated as VEGF_121_/rGel, was produced as previously described (9). Hypericin was obtained from Sigma-Aldrich (St Louis, MO, USA) while Anti-mouse CTLA-4 (CD152) and anti-mouse CD8α were obtained from BioxCell (Lebanon, NH, USA). *In vitro* TPCS_2a_ light exposure was performed with a LumiSource TM lamp (PCI Biotech AS) while TPCS_2a_ *in vivo* light exposure was done with a 652nm diode laser (CeramOptec GmbH, Bonn, Germany). For Hypericin yellow irradiations were carried out with a custom-made lamp equipped with an array of 1W LEDs (18). The irradiance was measured to 0.9 mW cm^−2^ by a photodetector PH100-SiUV coupled to a calibrated optic power meter Gentec-EO SOLO2 (Gentec-EO, Quebec, Canada). The spectrum of the yellow lamp was measured by an irradiance-calibrated AvaSpec-2048x14-SPU2 FiberOptic Spectrometer (Avantes, Apeldoorn, The Netherlands) and peaked at approximately 590 nm. For more information see **Supplementary Material**.

### *In vitro* experimental design and evaluation of DAMP signaling

CT26.WT or MC38 cells were seeded and incubated with 0.6 µg/ml TPPS_2a_ for 18 h, washed and chased 4 hrs prior to light exposure with LumiSource for 1.5 min (low light dose) or 4 min (high light dose). For VEGF121/rGel-PCI cells were incubated with 10nM VEGF121/rGel the last hour of the 4 hrs chase, and the medium was changed immediately prior to light exposure. For Hypericin-PDT incubated with 500nM Hypericin for 18 hrs and the medium was changed immediately prior to light exposure for 1.5 min (low light dose) or 4.5 min (high light dose). For all photodynamic treatments cell viability was measured 48 hrs after light exposure using the (3-(4,5-dimethyl-2-thiazolyl)-2,5-diphenyl-2H-tetrazolium bromide) MTT assay as previously described (19). For evaluation of DAMPs medium was harvested from the treated cells and subjected to an Adenosine 5′-triphosphate (ATP) Bioluminescent Assay Kit (Sigma-Aldrich). HMGB1 and HSP90 release was assessed by Western blotting as previously described (20) using an αHSP90 antibody (#4877) from Cell Signaling Technologies (CST) (Danvers, MA, USA) (1:1000) and an αHMGB1 antibody (#ab18256) from Abcam Cambridge UK)(1:1000). For more details, see **Supplementary Material**.

### Fluorescence microscopy of DAMP signals post VEGF_121_/rGel-PCI

CT26WT cells were seeded on cover slips (No. 1014/10. Assistent, Sondheim, Germany) and treated with PDT or VEGF_121_/rGel-PCI as indicated. Cells were fixed in 4% paraformaldehyde (PFA) and stained with Phalloidin-iFluor 594 Reagent from Abcam (#ab176757 1:1000 in PBS) before they were stained with primary antibodies αHMGB1 (#ab18256, Abcam 1:1000) or αHSP90 (#4877 CST 1:100) over night at 4°C, incubated with secondary Goat αrabbit Alexa 488 antibody (#A11034, Life Technologies1:600 in 1%BSA), incubated 2 min with 0.6 µg/ml Hoechst 3325 (Sigma Aldrich), and mounted using ProLong Glass Antifade mountant (Thermo Fisher). The cells were subjected to microscopy using a LSM 880 Airyscan FAST confocal microscope equipped with an Airyscan detector and FAST options, Ar-laser multiline (405/458/488/514/561 and 633 nm and 20x NA 0.8 DIC II (Plan-Apochromat) and 63x NA 1.4 oil DIC III (Plan-Apochromat) objectives (Carl Zeiss AG, Oberkochen, Germany). The Zen blue software (Carl Zeiss AG) was used for image acquisition and processing. For more details, see **Supplementary Material**.

### Tumor allografts

CT26WT cells were injected (100 000 cells in 30µl PBS) s.c. on the left flank in Balb/c. MC38 cells were injected (500 000 cells in 30µl PBS) s.c. on the right side of the abdomen of C57BL/6 mice. The tumors, body weight and general appearance was measured and evaluated 2-3 times per week. Tumor volume (V) was measured using a digital caliper and the formula V = (WxWxL)/2 where W is the width and L the length of the tumor. The protocol was designed with two endpoints; tumor size 1000 mm^3^ and/or weight loss > 20%. Animals were euthanized by cervical dislocation when reaching one of the endpoints.

### *In vivo* experimental design and methods

**Figure 2:**
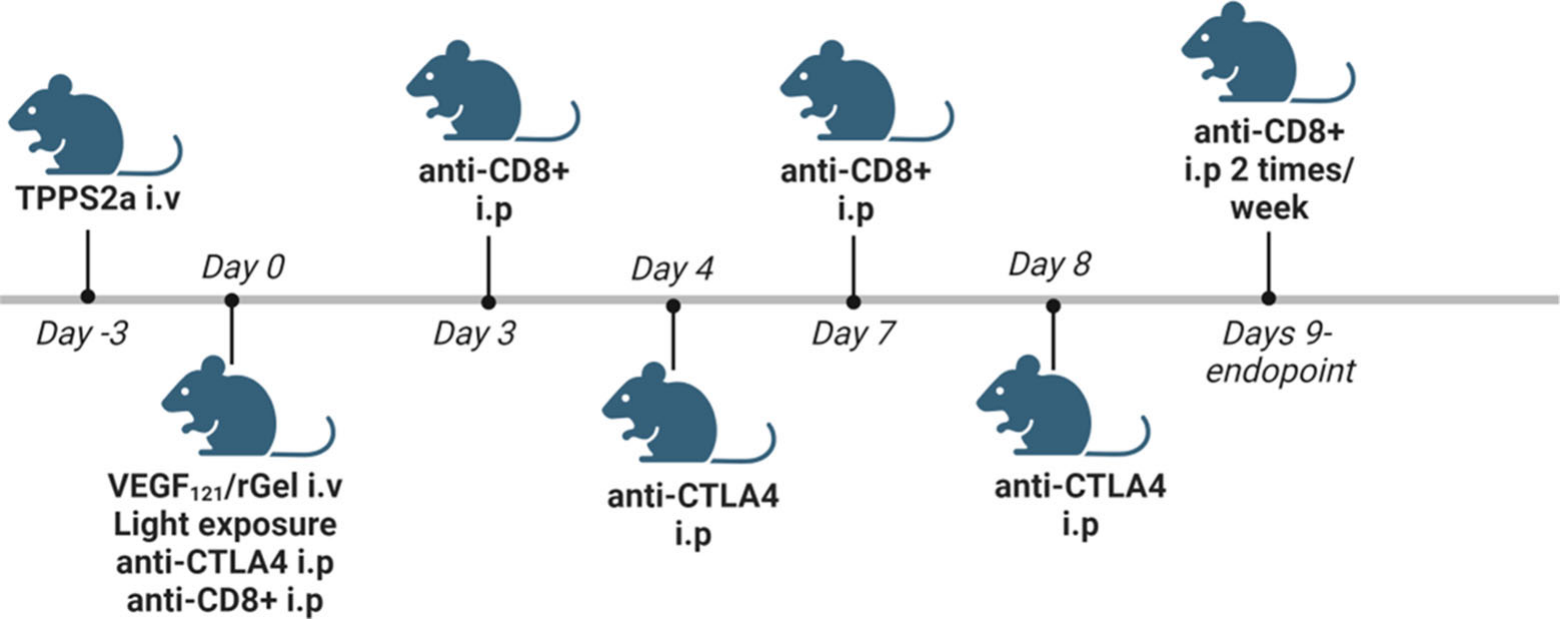
Schematic *in vivo* treatment schedule for VEGF_121_/rGel-PCI enhanced αCTLA4, including experiments with αCD8+ depletion. Created with BioRender.com.

TPCS_2a_ was administrated i.v. through the lateral tail vein 0.1 mg/mouse 4 days after tumor inoculation. Seventy-two hrs post TPCS_2a_ administration, 0.01 mg VEGF_121_/rGel in 100 µl PBS was administrated i.v. through the lateral tail vein and the animals were left 6 hrs prior to light exposure with the diode laser at a total dose of 15 J/cm^2^ for Balb/c mice or 10 J/cm^2^ for C57BL/6 mice. The animals were covered with aluminum foil during light exposure except from the tumor area and an additional 2-3 mm rim. αCTLA4 antibody was injected in 50µl 0.9% NaCl i.p. 3 times (directly after light exposure, day 4 and day 8) at a total dose of 15 µg (5 µg/injection), 50 µg (16.5 µg/injection) and 150 µg (50 µg/injection). αCD8^+^ antibody was injected 200 µg in 50 µl 0.9% NaCl i.p. directly after light exposure, on day 3, 7 and then twice/week until endpoint was reached. Animals receiving TPCS_2a_ were kept under subdued light for one week after administration to avoid phototoxicity. A schematic illustration of the treatment protocol is provided in **Figure 2**. The number of animals in each treatment group is indicated in **Figure S6C** for the CT26WT allograft and **Figure S8E** for MC38 (3. column in each table).

### Statistics

All *in vitro* experiments were reproduced at least twice and the data presented are the average of three independent experiments. Evaluation of significant differences between two *in vitro* treatments was done by two-sided student t-tests. A pared t-test was used when indicated to correct for differences in PDT-induced cytotoxicity between the three replicates. The *in vivo* experiments were performed with at least 5 animals per treatment group. For *in vivo* data, one-way ANOVA test and Holm-Sidak *post-hoc* tests were performed to evaluate significant differences between all groups in a data set. Statistical differences in time to reach endpoint were evaluated by pairwise log-rank analysis and subsequent Holm-Sidak *post hoc* tests. Sigmaplot version 14.5 (Systat Software Inc, San Jose, Ca, USA) was used for all the statistical analysis and p ≤ 0.05 was considered statistically significant.

## Results

### VEGF_121_/rGel-PCI induces enhanced DAMP signaling

**Figure 3:**
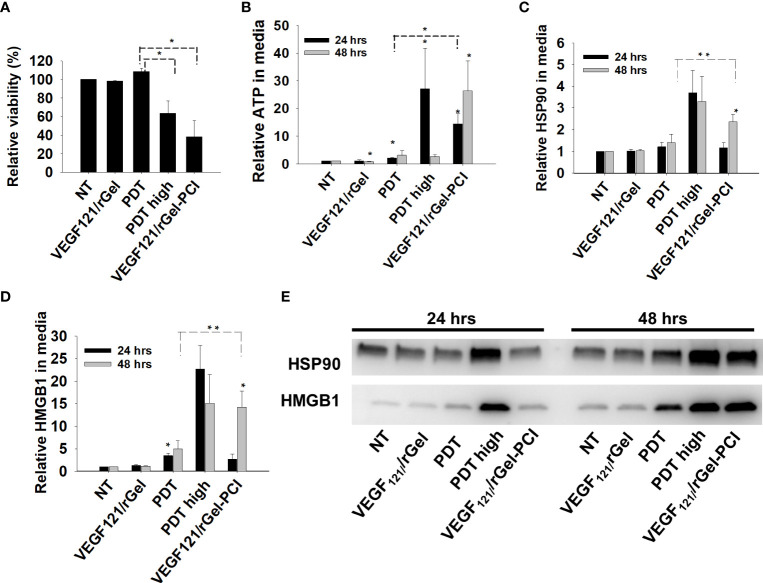
VEGF_121_/rGel-PCI induces secretion of DAMP signals from CT26 cells. **(A)** Relative cell viability (MTT) 48 hrs post VEGF_121_/rGel-PCI with indicated controls. **(B)** Normalized ATP secretion (bioluminescence assay) 24 and 48 hrs post VEGF/Gel-PCI with indicated controls. **(C, D)** HSP90 and HMGB1 secretion (quantification of western blots) 24 and 48 hrs post VEGF_121_/rGel-PCI with indicated controls. The graphs show averages of 3 independent experiments with error bars indicating SD. Bar labeled with * indicate p < 0.05 as compared to non-treated control (NT) (t-test). Significance between two treatments is indicated with * and dotted line (t-test). ** indicate significance with paired t-test. **(E)** Representative western blots of 3 independent experiments of HSP90 and MHGB1 in cell media harvested 24 and 48 hrs post treatment. VEGF_121_/rGel-PCI was performed with the same light dose as used with PDT while PDT_high_ was executed at a higher light dose.

PDT-induced enhancement of immunogenicity has previously been related to ICD involving induction and secretion of (DAMPs) such as heat shock protein (HSP) 70, HSP 90, calreticulin, HMGB1 and ATP. ICD has further been shown to recruit and activate APCs (21, 22). We here investigated if VEGF_121_/rGel-PCI could increase DAMP signaling as compared to PDT (TPCS_2a_+light). DAMP signaling was evaluated following VEGF_121_/rGel-PCI in CT26 cells at a dose reducing the viability to ~40% (**Figure 3A**). This light dose did not affect viability when combined with the photosensitizer in the absence of VEGF_121_/rGel (PDT) (**Figure 3A**). PDT with a high light dose was also investigated as a control, reducing the viability to ~60%, presented as PDT_high_. This control was included to evaluate PDT and PCI at similar viability level.

Secretion of ATP, HSP90 and HMGB1 into the CT26 cell media was evaluated at 24 and 48 hrs after treatment. VEGF_121_/rGel-PCI-induced ATP secretion was strongly enhanced as compared to PDT 24 hrs after the same dose of light (**Figure 3B**). Forty-eight hrs after treatment the amount of ATP secreted to the medium after VEGF_121_/rGel-PCI appeared much higher (2,5-18-fold) than in the paired PDT cohort, but due to the large spread the difference was only at the boarder of significant (p=0,074, paired t-test) (**Figure 3B**). Furthermore, the kinetics of ATP secretion was different between VEGF_121_/rGel-PCI and PDT, where the ATP release was increased between 24 and 48 hrs after VEGF_121_/rGel-PCI, while PDT did not induce a substantial ATP release and PDT_high_ peaked 24 hrs after treatment (**Figure 3B**). The amount of ATP measured in media from VEGF_121_/rGel-PCI treated cells 48 hrs after treatment was comparable to that measured in media from PDT_high_ 24 hrs after treatment (p=0.973). The extracellular half-life of ATP in our cell system is probably relatively short and previously reported between 40 min and 3 hrs in similar systems (23). Thus, the different kinetics of ATP secretion between TPCS_2a_-light and VEGF_121_/rGel-PCI observed here probably reflects different times to reach maximal ATP secretion.

The secretion of HSP90 and HMGB1 after PDT and VEGF_121_/rGel-PCI followed a similar pattern as for ATP, minimal secretion after PDT alone, while in combination with VEGF_121_/rGel, i.e. PCI, a substantial secretion was seen 48 hrs after treatment. PDT with a light dose inducing a similar treatment effect as after PCI,i.e. PDT_high_ resulted in a similar level of HSP90 and HMGB1 secretion 24 hrs earlier (**Figures 3C–E**). The half-life of extracellular HSP90 and HMGB1 in cell media is probably several days, and the lack of increase in HSP90 and HMGB1 release from 24 to 48 hrs after PDT therefore indicate no increased release during this time frame. This was in contrast to VEGF_121_/rGel-PCI where a significant increase in HSP90 and HMGB1 release was found between 24 and 48 hrs, indicating a stronger and more prolonged release as compared to PDT (**Figures 3C–E**).

**Figure 4:**
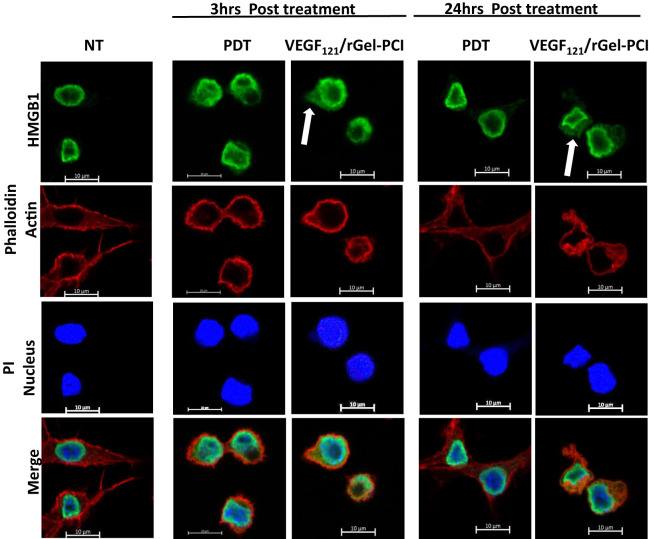
Intracellular localization of HMGB1 post VEGF_121_/rGel-PCI. Fluorescence images of CT26 cells showing intracellular localization of HMGB1 (green) 3 and 24 hrs post VEGF_121_/rGel-PCI with indicated controls. Fluorescence from DAPI (blue), staining the nucleus, and Phalloidin (red), staining the actin filaments, is included for orientation. Yellow color indicates co-localization between green and red. Bar: 10μm. VEGF_121_/rGel-PCI was performed with the low light dose. All images are representative for 3 independent experiments.

HSP90 and HMGB1 were further evaluated by fluorescence microscopy 3 and 24 hrs after VEGF_121_/rGel-PCI and PDT at both light doses (**Figures 4**, **S1**). Non-treated cells showed fluorescence from HSP90 localized both in the nucleus and in the cytosol. After light exposure the cells were rounded up, but showed no difference in HSP90 localization (**Figure S1A**). HMGB1 was mainly detected close to the nuclear membrane in non-treated as well as PDT treated cells (both light doses) at both time points (**Figures 4**, **S1B**). VEGF_121_/rGel-PCI induced, however, cytoplasmic localization of HMGB1 at both time points (**Figures 4**, **S1B**).

**Figure 5:**
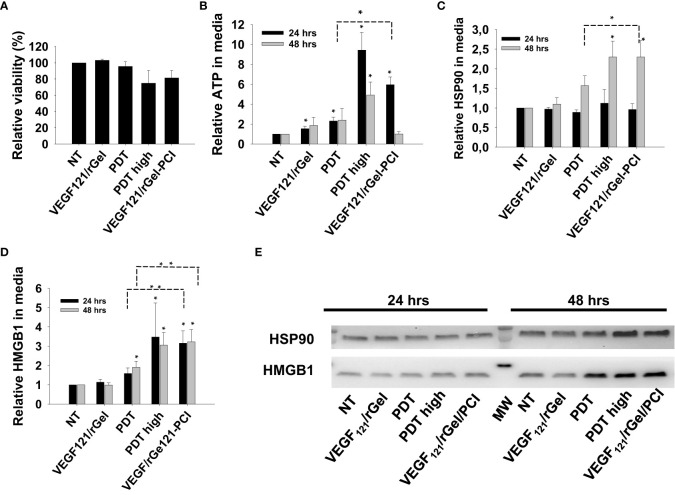
VEGF_121_/rGel-PCI induces secretion of DAMP signals from MC38 cells. **(A)** Relative cell viability (MTT) 48 hrs post VEGF_121_/rGel-PCI with indicated controls. **(B)** Normalized ATP secretion (bioluminescence assay) 24 and 48 hrs post VEGF/Gel-PCI with indicated controls. **(C, D)** HSP90 and HMGB1 secretion (quantification of western blots) 24 and 48 hrs post VEGF_121_/rGel-PCI with indicated controls. The graphs show an average of 3 independent experiments with error bars indicating SD. Bar labeled with * indicate p < 0.05 as compared to non-treated control (NT) (t-test). Significance between two treatments is indicated with * and dotted line (t-test). ** indicate significance with paired t-test. **(E)** representative western blots of 3 independent experiments of HSP90 and MHGB1 in cell media harvested 24 and 48 hrs post treatment. VEGF_121_/rGel-PCI was performed with the same light dose as used with PDT while PDT_high_ was executed at a higher light dose.

We further verified our DAMP signaling results in MC38 cells with a light dose reducing the viability to 80% for VEGF_121_/rGel-PCI, 95% for PDT and 75% for PDT_high_ (**Figure 5A**). Also in MC38 cells we observed a stronger DAMP signaling following VEGF_121_/rGel-PCI than after PDT, but comparable to PDT_high_ (**Figures 5B–E**). In contrast to CT26 cells, the kinetics of DAMP release did not differ between PDT, PDT_high_ and VEGF_121_/rGel-PCI. Maximum ATP and HMGB1 release was detected at 24 hrs after both PDT_high_ and VEGF_121_/rGel-PCI (**Figures 5B, D**) while the release of HSP90 was slower and only detected 48 hrs after treatment (**Figure 5C**). The release of HSP90 in the MC38 cell line was also slower than in the CT26 cells (**Figures 3C**, **5C**).

### Photoactivation of TPCS_2a_ induce DAMP signaling comparable to that of hypericin

**Figure 6:**
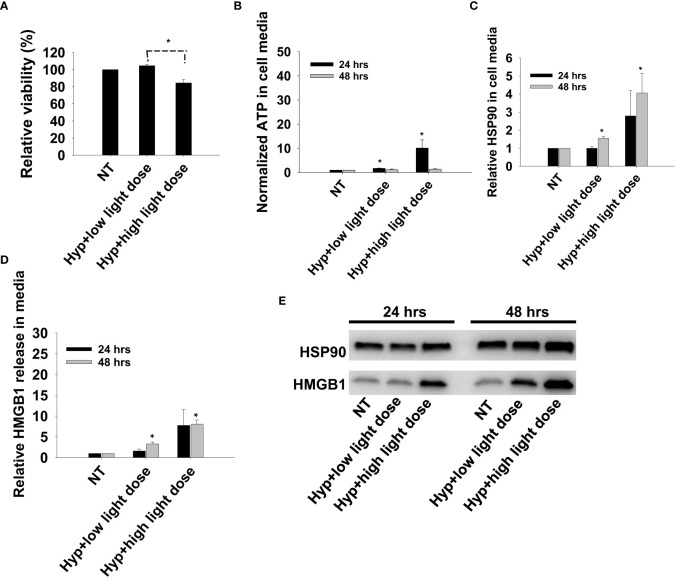
Photoactivation of hypericin induces secretion of DAMP signals from CT26 cells. **(A)** Relative cell viability (MTT) 48 hrs post photoactivation of hypericin at two light doses (high and low). **(B)** Normalized ATP secretion (bioluminescence assay) 24 and 48 hrs post photoactivation of hypericin at high and low light dose. **(C, D)** HSP90 and HMGB1 secretion (quantification of western blots) 24 and 48 hrs post photoactivation of hypericin at two light doses. The graphs show an average of 3 independent experiments with error bars indicating SD. Bar labeled with * indicate p < 0.05 as compared to non-treated control (NT) (t-test). Significance between two treatments is indicated with * and dotted line (t-test). **(E)** representative western blots of 3 independent experiments of HSP90 and MHGB1 in cell media harvested 24 and 48 hrs post treatment.

Previous reports on DAMP signaling following PDT has been conducted with other photosensitizers and hypericin has been argued as a highly efficient photosensitizer for light induced DAMP signaling (21). To our knowledge, this is the first report on TPCS_2a_-PDT induced DAMP signaling, and Hypericin-PDT was included as a control in CT26 cells to compare the magnitude of signal with these highly different photosensitizers. Photoactivation of hypericin was done at two light doses, one sub-lethal and one reducing the viability to 80% (**Figure 6A**). An increase in secretion of all three factors, ATP, HMGB1 and HSP90, was observed after light-activation of hypericin (**Figures 6B–E**). The magnitude and kinetics of DAMP signaling post hypericin-PDT in CT26 cells was found similar as observed for TPCS_2a_+light (**Figures 3**, **6**).

### VEGF_121_/rGel-PCI in CT26 tumors enhance CD8^+^-mediated αCTLA4 efficacy in early phase

**Figure 7:**
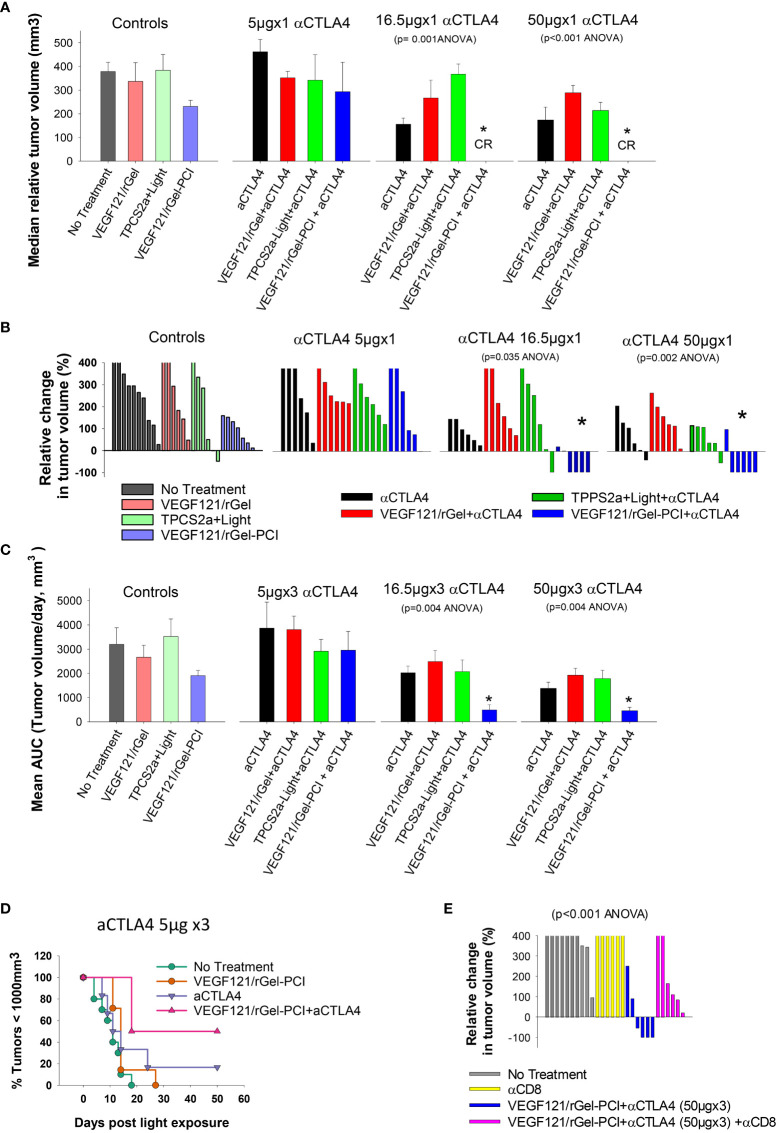
VEGF_121_/rGel-PCI enhances αCTLA-4 treatment in CT26 tumors dependent on CD8^+^ T-cells. **(A)** Median relative tumor volume at day 4 as compared to day 0 in each treatment group. **(B)** Relative change in tumor volume from day 0 to day 4 for each individual animal within the treatment groups (The tumor volume distribution is shown in **Figure S4**). **(C)** Average AUC integrated from day 0 to day 16 for each treatment group. The panels represent increasing doses of αCTLA-4 antibody. Error bars: SE. **(D)** Kaplan-Meier plots illustrating overall treatment response of VEGF_121_/rGel-PCI +αCTLA4 (5µgx3) as measured by tumors with volume < 1000mm^3^ with indicated controls. **(E)** Relative change in tumor volume from day 0 to day 9 for each individual animal within the indicated treatment groups. αCTLA-4 antibody was used at a total dose of 150µg (50µgx3). Within the figure panels, significant difference between the treatment groups is evaluated by ANOVA and the p value is indicated when significant. * indicate significant difference (p<0.05) from the other groups.

It was evaluated if VEGF_121_/rGel-PCI could potentiate the efficacy of αCTLA4 treatment in two murine allografts, the CT26 and MC38 model. VEGF_121_/rGel-PCI as a neoadjuvant to αCTLA4 treatment in CT26 tumors was evaluated at 3 doses (accumulated dose) of αCTLA4 antibody; 15μg 50μg and 150μg per mouse split into 3 injections with a time interval of 4 days where the first injection was administered immediately after the light exposure (**Figure 2**). The immediate effect, i.e. 4 days after treatment, was an apparent complete response by combining VEGF_121_/rGel-PCI and the 2 highest doses of αCTLA4 (16.5 and 50 μg, **Figure 7A**). During these 4 days the control tumor volume increased almost 4-fold and in all the other treatment regimens the tumors also increased 2-4 fold in volume (**Figure 7A**). The reduction in CT26 tumor volume following VEGF_121_/rGel-PCI enhanced αCTLA4, as measured on day 4 relative to initial volume (**Figure 7B**), was found significant (p < 0.05, one way ANOVA test) after+ 16.5µgx1 and 50µgx1 αCTLA4, but not 5µgx1. Subsequently, all pairwise multiple comparison (Hom-Sidak method) identified VEGF_121_/rGel-PCI enhanced αCTLA4 treatment as significantly more efficient than all other treatments at 50µgx1 αCTLA4 and from VEGF_121_/rGel+ αCTLA4 at the 16.5µg dose (**Figure 7B**). αCTLA4-monotherapy induced CT26 tumor growth delay in a dose dependent manner as seen from the decreased median tumor volume at day 4 (**Figure 7A**) as well as by change in tumor volume of each individual tumor (**Figures 7B**, **S3**) and median time to reach endpoint (S6B). The tumor volume distribution at day 4, 7, 9 and 11 is shown in **Figures S4**, **S5** for each treatment group. In all the αCTLA4 monotherapy groups the tumor continued to grow the first 4 days after treatment, but following 2 additional αCTLA4 administrations the tumors decreased in volume resulting in 67% and 83% CR in the 50µg and 150 µg groups, respectively, as measured at day 100 (**Figure S6C**). VEGF_121_/rGel-PCI was executed at a dose previously indicated to induce immune-mediated tumor eradication in 50% of CT26.CL25 (β-Gal transfected) bearing BALB/c mice (11). However, no complete responses were observed here in the CT26 wild type model. A reduced CT26 growth rate was notably observed in the VEGF_121_/rGel-PCI treated animals causing a smaller increase in tumor volume the first 7-9 days after treatment (**Figure S3A**). This effect was however transient and no difference in time to reach 1000 mm^3^ were observed between VEGF_121_/rGel-PCI and the other controls (**Figures S6A, B**).

The fraction of CT26 tumors with reduced volume, relative to initial volumes, was also evaluated as a function of time after treatment (**Figure S2**). The first two weeks after treatment initiation VEGF_121_/rGel-PCI-enhanced αCTLA4 treatment (50µg and 150µg) reduced the tumor volume in 66-83% of the animals (**Figure S2** blue lines, two right panels). The integrated CT26 tumor burden for the first 16 days following VEGF_121_/rGel-enhanced αCTLA4 (50 µg and 150 µg) was also significantly reduced as compared to all other treatment regimens (**Figure 7C**, the two right panels). The overall treatment outcome of αCTLA4 was increased using VEGF_121_/rGel-PCI as an adjuvant as shown with the 5µgx3 dosage (with minor effect as monotherapy) (**Figure 7D**).

In order to evaluate the impact of CD8^+^ T-cells on the treatment outcome αCD8^+^ antibodies was administered after light exposure. In CT26 tumors, the tumor reduction induced by VEGF_121_/rGel-PCI enhanced αCTLA4 was blocked in the presence of the αCD8^+^ antibody, and no animals in this combination group showed reduction in tumor volume when measured at day 9 (**Figure 7E**). Overall, all the CT26 tumors treated with VEGF_121_/rGel-PCI enhanced αCTLA4 (150µg) and CD8^+^ depletion reached the 1000m^3^ end point within 40 days after treatment initiation (**Figure S6D**). Furthermore, all the animals obtaining CR (regardless of treatment) showed long lasting immunity against CT26, as no tumors were detected upon CT26 rechallenge 100 days post treatment and at least 60 days follow up.

### VEGF_121_/rGel-PCI is highly tolerable in the CT26 model

In the CT26 model, non-treated animals had an increase (4.5%) in weight during the first 4 days (**Figure S7**). αCTLA4 monotherapy was highly tolerable and no difference in animal appearance or weight was found as compared to the controls throughout the experiment (data shown for Day 4). VEGF_121_/rGel-PCI caused a minor weight reduction (3.6%) at day 4 as compared to the day of treatment initiation, but the weight was restored to control levels already at day 7 (data not shown). Combining αCTLA4 with VEGF_121_/rGel-PCI caused a larger reduction (6.6%) in animal weight at day 4, although not significant different from VEGF121/rGel-PCI (**Figure S7**). This weight reduction was transient and restored at day 11 (**Figure S7**). In addition, all light exposed animals experienced swelling and redness in the light exposed area the first 3-5 days after light exposure, but no difference was observed between the groups. Thus, the tolerability of VEGF_121_/rGel-PCI-enhanced αCTLA4 treatment was found high and comparable to that of VEGF_121_/rGel-PCI in the CT26 model.

### VEGF_121_/rGel-PCI enhance CD8+ dependent efficacy of αCTLA4 treatment in MC38 allografts

**Figure 8:**
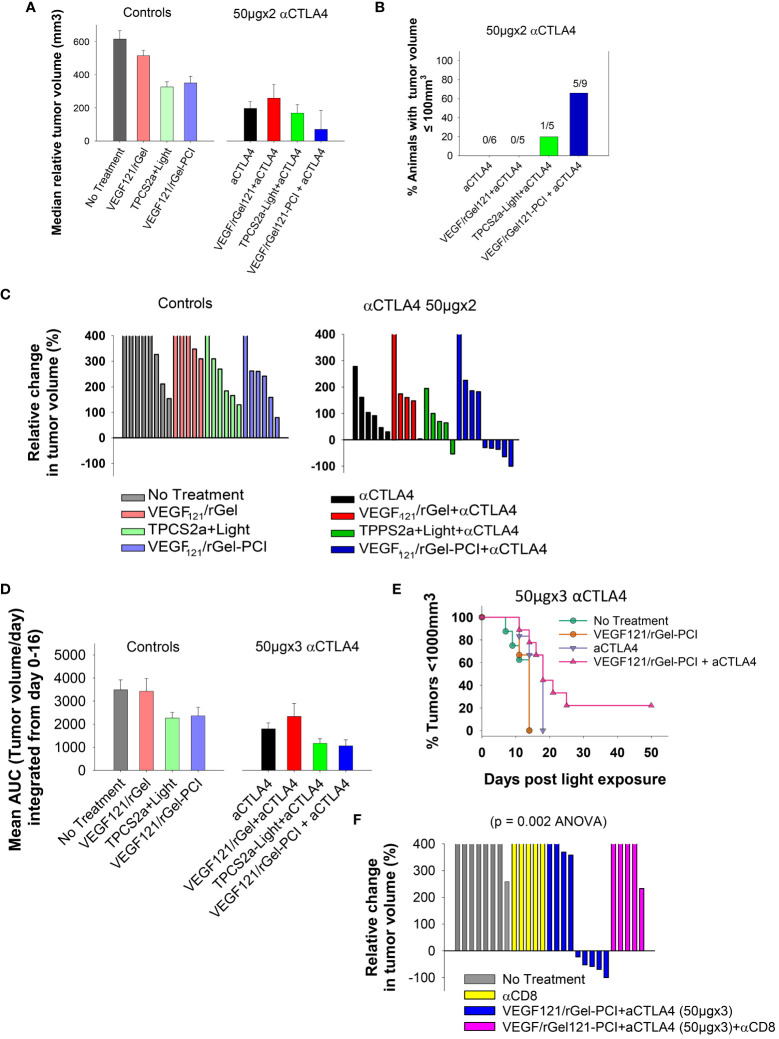
VEGF_121_/rGel-PCI enhances αCTLA-4 treatment in MC38 tumors. **(A)** Median relative tumor volume at day 7 as compared to day 0 in each treatment group. **(B)** Relative number of animals with decreased tumor size at day 7 as compared to day 0 in each treatment group. The numbers above the bars indicate number of animals with decreased size per total number of animals within the group. **(C)** Relative change in tumor volume from day 0 to day 7 for each individual animal within the treatment groups (The tumor volume distribution is shown in **Figures S9A, B**). The 2 panels represent 0 and 50µgx2 αCTLA-4 antibody. **(D)** Average AUC integrated form day 0 to day 16 for each treatment group. The panels represent 0 and 50µgx3 αCTLA-4 antibody. Error bars: SE. **(E)** Kaplan-Meier plots illustrating overall treatment response of VEGF_121_/rGel-PCI +αCTLA4 (50µgx3) as measured by tumors with volume < 1000mm^3^ with indicated controls. **(F)** Relative change in tumor volume from day 0 to day 9 for each individual animal within the indicated treatment groups. αCTLA-4 antibody was used at 50µgx3.

Although not as pronounced, similar results as obtained with the CT26 model were observed with the MC38 model. VEGF_121_/rGel-PCI and αCTLA4 (50µgx2) as monotherapies both induced minor growth delay as compared to non-treated controls as measured as the relative median tumor volume at day 7 (**Figure 8A**), although not significant (ANOVA). Nevertheless, VEGF_121_/rGel-PCI enhanced αCTLA4 induced an immediate reduction in MC38 tumor volume in 5/9 animals as measured at day 7 (**Figures 8B, C**), and the effect was sustained and still pronounced at day 11 where 4/9 animals still had a reduced tumor volume and 2/9 where in CR (**Figures S9B, E**). In the MC38 model, the combination of TPCS_2a_, light and αCTLA4 also seemed to induce an immediate reduction in tumor volume as measured at day 4 when 4/5 animals had reduced tumor volume (**Figure S9B**). However, this effect was more transient and only 1/5 animals showed reduced tumor volume when measured at day 7 (**Figure 8B**). The tumor volume distribution at day 4, 7, 9 and 11 for each treatment group is shown in **Figure S10**. Evaluation of the relative amount of MC38 tumors with reduced volume in the treatment groups as a function of time reviled that VEGF_121_/rGel-PCI enhanced αCTLA4 (50µgx3) in the MC38 model reduced the tumor volume in > 50% of animals within two weeks after treatment as compared to all the other treatment groups (**Figure S8**). The accumulated tumor burden 0-16 days following VEGF_121_/rGel enhanced αCTLA4 150µg was also reduced as compared to the controls, however, not significant and similar to that observed following TPCS_2a_+light+ αCTLA4 (**Figure 8D**). As observed in the CT26 model the overall treatment outcome of αCTLA4 was increased using VEGF121/rGel-PCI as an adjuvant (**Figure 8E**). Furthermore, CD8^+^ depletion inhibited VEGF_121_/rGel-PCI enhanced αCTLA4-induced MC38 tumor reduction, and no animals experienced reduction in tumor volume following VEGF121/rGel-PCI enhanced αCTLA4 in the presence of an αCD8^+^ antibody (**Figures 8F**, **S9F**).

### VEGF_121_/rGel-PCI is highly tolerable in the MC38 model

No difference in weight was observed in any of the treatment groups within the first 4 days after treatment of the MC38 allografts. Weight increase was observed in all the groups from day 11, including the VEGF_121_/rGel-PCI + αCTLA4 treated animals (**Figure S11**). All light exposed animals experienced swelling and redness in light exposed area the first 3-5 days after light exposure, but no difference was observed between the groups. Thus, the tolerability of VEGF_121_/rGel-PCI enhanced αCTLA4 was found high and comparable to that of VEGF_121_/rGel-PCI monotherapy also in the MC38 model.

## Discussion/conclusion

Immune Checkpoint blockades represent a game changer in cancer therapy that opens the possibility for cures in previously non-curable cancers including metastatic melanoma (24). Nevertheless, suboptimal efficacy as well as toxicity represents major challenges for ICIs, and adjuvants that can enhance the efficacy without compromising toxicity is highly warranted. Thus, there is a need to enhance the efficacy of ICI, not only to increase the response rate in approved indications but also to obtain efficacy in currently non-responsive cancers. Certain chemotherapeutic drugs as well as molecular inhibitors and radiotherapy have shown encouraging results as adjuvants in several clinical trials, however, little is still known as to how we can best potentiate the efficacy of ICIs with respect to choice of adjuvant, dosage and treatment schedule.

ICIs are proposed to benefit from adjuvant treatment inducing ICD (25, 26). Several studies have demonstrated PDT as an highly efficient inducer of ICD, and PDT has been shown as an efficient ICI enhancer in several preclinical studies using a large variety of photosensitizers (27–31). The ability of a photosensitizer to induce ICD is dependent on its chemical properties and subsequent intracellular localization pattern where localization to endoplasmic reticulum (ER) is proposed superior for ICD induction (21, 32–34), as well as the treatment dose. In the present study we have investigated ICD following PDT with the clinical relevant amphiphilic photosensitizer TPCS_2a_. TPCS_2a_ is well known to primary localize to endosomes and lysosomes within the cells (35). Furthermore, TPPS_2a_, a photosensitizer structurally highly similar to TPCS_2a_, is indicated to translocate to ER during light exposure with subsequent photochemical damage to this organelle (36). The present report shows for the first time that light activation of TPCS_2a_ induces DAMP signaling similar to that of Hypericin at comparable and low dosages with respect to cytotoxicity. ATP-, HSP90- and HMGB1- secretion was here found post TPCS_2a_-PDT in both CT26WT and MC38 cells. Hypericin-PDT is reported as a strong inducer of ICD (32) and thus the current results indicate thatTPCS2a-PDT is also a strong inducer of ICD. The strong enhancement of ICD by PCI-activation of VEGF_121_/rGel indicate that VEGF_121_/rGel is also inducing ICD. Altogether, it is here shown that light activation of the clinical relevant photosensitizer TPCS_2a_ induce efficient DAMP signaling. We also investigated both membrane translocation and cellular secretion of another DAMP marker, calreticulin (CRT). However, in contrast to what has been published before (21), we could not detect any reproducible change in cellular localization of CRT or secretion of CRT upon photochemical treatment with either photosensitizer.

TPCS_2a_ was developed as a photosensitizer for the drug delivery system PCI, where the photochemical reaction is used to destabilize the endo/lysosomal membrane, and thereby release the drugs entrapped inside (35). We have previously reported on both vascular-and immune-mediated efficacy of the targeted toxin VEGF121/rGel when delivered with PCI, and indicated this as stronger than provided by TPCS_2a_ and light only (TPCS_2a_-PDT) (11). This is in agreement with the present *in vitro* data demonstrating enhanced DAMP signaling of VEGF_121_/rGel-PCI as compared to TPCS_2a_-PDT. ICD of type I ribosome inactivating protein toxins (RIPs) such as gelonin as well as the type II RIPs have never been reported although immunotoxins based on the bacterial pseudomonas exotoxin (PE) has been reported to induce ICD (37–39). As both gelonin and Pseudomonas exotoxin exert cytotoxicity by inhibiting protein synthesis we find it likely that the enhanced DAMP signaling of VEGF_121_/rGel PCI as compared to PDT is mediated by gelonin. In line with this, evaluating 50 000 agents for ICI induction Humeau et al. found that most ICI inducers cause an DNA-to-RNA translation and subsequently also inhibit RNA-to-protein translation (40). Furthermore, in our previous report on VEGF121/rGel-PCI we showed a striking difference between complete remissions in immunocompetent Balb/c (67%) as compared to immunodeficient nude mice (0%) using the CT26.CL25 model. The ability of VEGF_121_/rGel-PCI to induce immune-mediated efficacy was further indicated here using the CT26WT model where no CR was detected as compared to what was published in the more immunogenic CT26.CL25 model (11). Thus, VEGF_121_/rGel-PCI induces a strong immune-response that holds promise as an ICI enhancer. Indeed VEGF_121_/rGel PCI was here demonstrated as an αCTLA4 enhancer both in the CT26WT model and in the MC38 model. The effect of the enhancement was most pronounced in the CT26 model at early time points before the αCTLA4 treatment was finalized and before significant tumor growth delay was observed from αCTLA monotherapy. CTLA4 function early in the immune-response, when naïve T-cells are activated by antigen presenting cells, but is also expressed on regulatory T-cells. Binding of CTLA-4 to CD80/CD85 on antigen-presenting cells inhibits activation and maturation of the T-cells. The response to αCTLA-4 monotherapy in the CT26 model was here observed ~10 days after the first αCTLA-4 injection. The effect of VEGF_121_/rGel-PCI enhanced αCTLA-4 treatment was however much more rapid and CRs were detected already 4 days after the first αCTLA-4 injection. The rapid response to VEGF_121_/rGel-PCI enhanced αCTLA-4 was dependent not only on the activation of the photosensitizer (PDT) but also on photochemical activation of VEGF_121_/rGel as shown by the control groups. VEGF_121_/rGel-PCI exerts its activity on both VEGFR1 and VEGFR2 expressing cells (11) and it is possible that VEGF_121_/rGel-PCI here target and kill a fraction of cells which enable a more rapid response to αCTLA-4 treatment. With respect to this, VEGFR2 has by others been shown overexpressed on both T_regs_ and Myeloid-derived suppressor cells (MDSC) and the small molecular VEGFR inhibitor Sunitinib has been shown to decrease the population of both T_Regs_ and MDCS (41). Nevertheless, even though the combination effect was most pronounced at early time points, VEGF_121_/rGel-PCI as an adjuvant was also shown to increase the overall treatment response in both models.

Combination of photosensitizer and light as utilized in both PDT and PCI induce a considerable inflammation and also enhance tumor immunogenicity (42). We here aimed to test if PCI with a targeting toxin was superior to PDT with respect to enhancing ICI efficacy. We selected PDT and PCI doses inducing only minor growth delay and experienced that PCI, but not PDT stimulated ICI enhancement. Although not investigated here, TPCS_2a_-PDT at increased dosage would probably induce ICI enhancement. The *in vitro* data, showing enhanced DAMP signaling of high dose PDT as compared to low dose PDT supports this hypothesis. Nevertheless, VEGF_121_/rGel-PCI is here shown as a better αCTLA-4 enhancer as compared to TPPCS_2a_-PDT. In conclusion VEGF_121_/rGel-PCI enhanced αCTLA4 is here shown to induce an immediate CD8^+^ dependent tumor eradication with impact on overall treatment response as compared to αCTLA monotherapy. The mechanisms behind this rapid efficacy were not addressed in this report and warrants further investigation to elucidate future ICI adjuvants.

## Data availability statement

The raw data supporting the conclusions of this article will be made available by the authors, without undue reservation.

## Ethics statement

The animal study was approved by Forsøksdyrforvaltningens tilsyns- og søknadssystem, Mattilsynet, Norway. The study was conducted in accordance with the local legislation and institutional requirements.

## Author contributions

AL: Methodology, Writing – original draft, Data curation, Formal analysis, Investigation. KB: Formal analysis, Investigation, Methodology, Validation, Writing – review & editing. AW: Methodology, Writing – original draft, Conceptualization, Funding acquisition, Project administration, Supervision.
