# Corrigendum: Light-enhanced VEGF_121_/rGel induce immunogenic cell death and increase the antitumor activity of αCTLA4 treatment

**DOI:** 10.3389/fimmu.2023.1359973

**Published:** 2024-01-09

**Authors:** Ane Sager Longva, Kristian Berg, Anette Weyergang

**Affiliations:** Department of Radiation Biology, Institute for Cancer Research, Norwegian Radium Hospital, Oslo University Hospital, Oslo, Norway

**Keywords:** immune check point inhibitor (ICI), photodynamic therapy, photochemical internalization (PCI), vascular targeting, immunogenic cell death (ICD), targeted toxin, vascular endothelial growth factor

In the published article, there was an error in **Figure 3** as published. 3C has wrongly also been inserted as 3D resulting in 3C showing the same figure as 3C. The figure legend is correct. The corrected **Figure 3** appear below.

**Figure 3:**
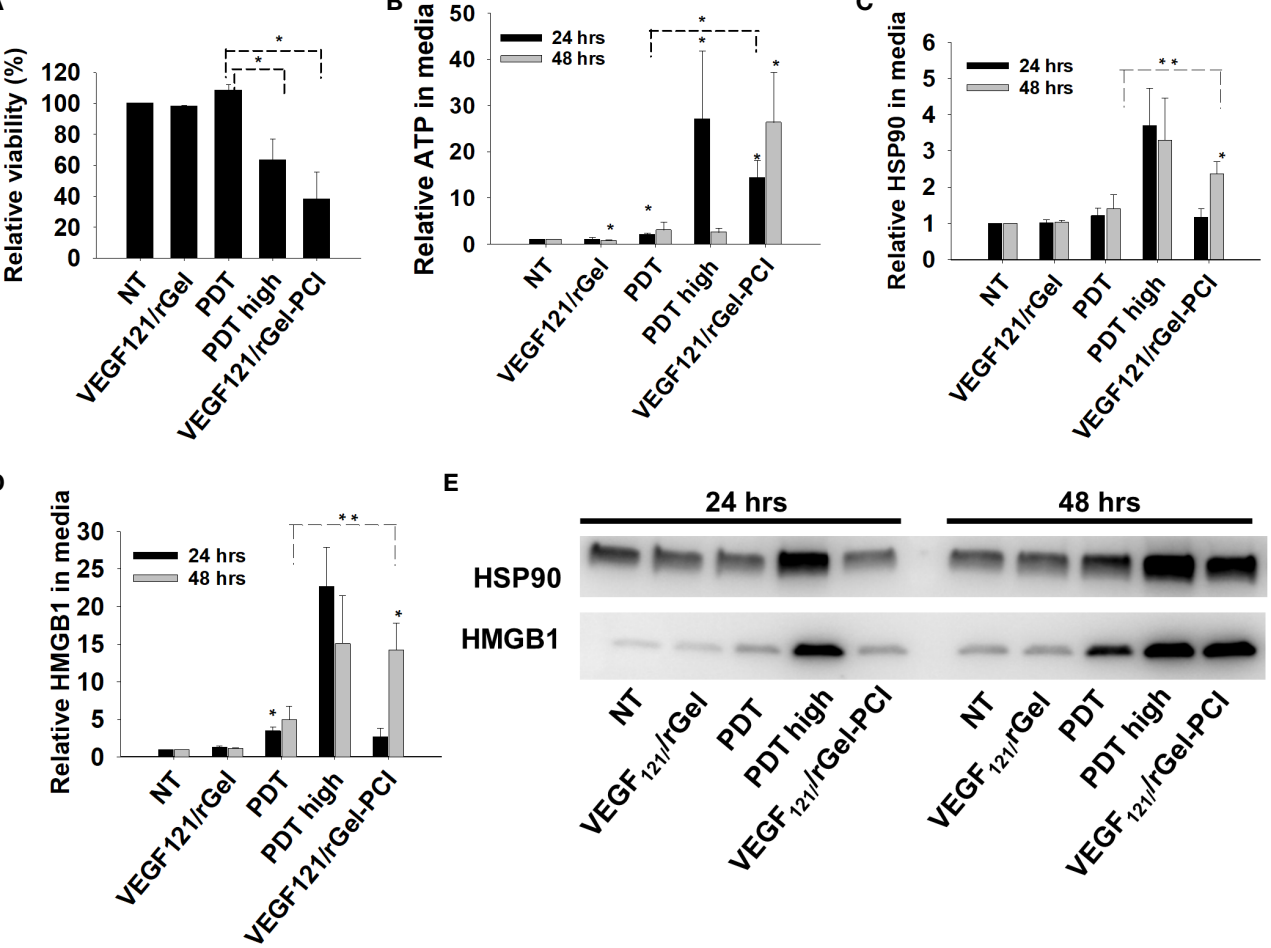
VEGF_121_/rGel-PCI induces secretion of DAMP signals from CT26 cells. **(A)** Relative cell viability (MTT) 48 hrs post VEGF_121_/rGel-PCI with indicated controls. **(B)** Normalized ATP secretion (bioluminescence assay) 24 and 48 hrs post VEGF/Gel-PCI with indicated controls. **(C, D)** HSP90 and HMGB1 secretion (quantification of western blots) 24 and 48 hrs post VEGF_121_/rGel-PCI with indicated controls. The graphs show averages of 3 independent experiments with error bars indicating SD. Bar labeled with * indicate p < 0.05 as compared to non-treated control (NT) (t-test). Significance between two treatments is indicated with * and dotted line (t-test). ** indicate significance with paired t-test. **(E)** Representative western blots of 3 independent experiments of HSP90 and MHGB1 in cell media harvested 24 and 48 hrs post treatment. VEGF_121_/rGel-PCI was performed with the same light dose as used with PDT while PDT_high_ was executed at a higher light dose..

The authors apologize for this error and state that this does not change the scientific conclusions of the article in any way. The original article has been updated.

