# Supplementary material for: Light-enhanced VEGF121/rGel induce immunogenic cell death and increase the antitumor activity of αCTLA4 treatment
Source: Front Immunol. 2023 Dec 19;14:1278000. doi: 10.3389/fimmu.2023.1278000 (PMC10762878; doi:10.3389/fimmu.2023.1278000)
Supplement: Supplementary file 1 [file DataSheet_1.pdf]

Supplementary Methods

Cell lines and culture conditions

Two murine colon cancer cell lines were used in this study. CT26.WT (CRL-2638) were obtained from ATCC (Manassas, VA, USA), maintained in RPMI (Sigma-Aldrich, St Louis, MO, USA) with 10% fetal bovine serum (FBS) (Thermo Fisher Scientific, Waltham, MA, USA), 100 U/ml penicillin and 100 µg/ml streptomycin (both from Sigma-Aldrich) . MC-38 (BE12-604F/U1) were obtained from Kerafast (Boston, MA ,USA) and maintained in DMEM (Lonza, Basel, Switzerland), supplied as the RPMI medium. Both cell lines were subcultured 2-3 times per week, grown at 37 °C in 75-cm^2 flasks in a humidified atmosphere containing 5% CO_2, used between passage numbers 5-25 and routinely checked for *Mycoplasma* infections.

Drugs and Chemicals

Meso-tetraphenyl chlorin disulphonate ($TPCS_{2a}$) (PCI Biotech AS, Oslo Norway) was dissolved at 0.35 mg/ml for *in vitro* use and 1mg/ml for in vivo use, in 3% Tween 80, 2.8% mannitol, 50mM Tris, pH 8.5 and kept protected from light at 4°C. $VEGF_{121}$/rGel, from now indicated as VEGF/rGel, was produced as previously described [9], but with an ÄKTA Avant automated chromatography system (GE Healthcare, North Richland Hills, Tx, USA) as recently reported [18]. Hypericin (Sigma-Aldrich) was dissolved at 2mM in dimethyl sulfoxide (DMSO) (Sigma-Adrich) and kept as aliquots at -20°C. Anti-mouse CTLA-4 (CD152) from now indicated as αCTLA4, and anti-mouse CD8α from now indicated as αCD8+, were obtained from BioxCell (Lebanon, NH, USA) and diluted in 0.9% NaCl prior to use.

Light sources

$TPCS_{2a}$ absorbs light both in the blue □$_{max}$=435nm) and red (□$_{max}$=652nm) wavelength region. *In vitro* $TPCS_{2a}$ light exposure was performed with a LumiSource TM lamp (PCI Biotech AS) consisting of four 18-W Osram L 18/67 light tubes which delivers blue light (λ=400-500nm, λ_{max} = 435nm) at a fluence rate of 7.1-9.6mW/cm^2. For $TPCS_{2a}$ *in vivo* light exposure with a 652nm diode laser (CeramOptec GmbH, Bonn, Germany) at an irradiance of 90 mW/cm^2 was used. For Hypericin a custom made lamp with 1mW LEDs delivering yellow light (λ=550-625nm, λ_{max} = 590nm) at a fluence rate of 0.9mW/cm^2 was used.

***In vitro* experimental design and evaluation of DAMP signaling**

CT26.WT or MC38 cells were seeded in 6-well plates at 60 000 cells/well or 40 000 cells/well respectively. For $TPCS_{2a}$-PDT or VEGF/rGel-PCI the cells were left to attached for 5 hrs followed by incubation with 0.6 µg/ml $TPPS_{2a}$ for 18 h. The cells were then washed once with medium and chased 4 hrs prior to light exposure with LumiSource for 1.5 min (low light dose) or 4 min (high light dose). For VEGF/rGel-PCI cells were incubated with 10nM VEGF/rGel the last hour of the 4 hrs chase, and the medium was changed immediately prior to light exposure. For Hypericin-PDT cells were left to attach 24 hrs after seeding followed by incubation with 500nM Hypericin for 18 hrs. The medium was changed immediately prior to light exposure using the yellow light source for 1.5 min (low light dose) or 4.5 min (high light dose). For all photodynamic treatments cell viability was measured 48 hrs after light exposure using the (3-(4,5-dimethyl-2-thiazolyl)-2,5-diphenyl-2H-tetrazolium bromide) MTT assay as previously described[19].

For evaluation of DAMPs secreted from the cells, medium was harvested from the treated cells 24 (400µl) and 48 (500µl) hrs after light exposure and subjected to an Adenosine 5′-triphosphate (ATP) Bioluminescent Assay Kit (Sigma-Aldrich) at each time point. Briefly, the medium was centrifuged 4 min

at 18.000 RCF 4°C, and the supernatant was transferred to new tubes and kept on ice until 100µl were used for ATP measurements according to the kit instructions. Luminescence was measured using a Spark 10M multifunctional microplate reader (Tecan, Männedorf Switzerland) in white 96 well plates using 50µl sample for each measurement and 50µl of the assay mix. What was left of the sample was stored at -80°C and used for evaluation of HMGB1 and HSP90 release by SDS-PAGE and Western blotting as previously described [18] using an αHSP90 antibody (#4877) from Cell Signaling Technologies (CST) (Danvers, MA, USA) (1:1000) and an αHMGB1 antibody (#ab18256) from Abcam Cambridge UK)(1:1000). HRP linked α-rabbit antibody from CST (#7074) were used as a secondary antibody, Supersignal West Dura Extended duration Substrate (Thermo Scientific, Waltham, Massachusetts, USA) was used as a detection reagent and a ChemiDoc™ densitometer (Bio-Rad, Hercules, California, USA) were used for the detection of protein bands on the membrane. ImageLab 4.1 (Bio-Rad) (software) was used for the quantification of protein expression.

Fluorescence Microscopy of DAMP signals post VEGF/rGel-PCI

CT26WT cells were seeded on cover slips (No. 1014/10. Assistent, Sondheim, Germany) in 48 well trays, 20 000 cells/well for non-treated cells, and 25 000 cells/well for treated cells, and left to attach overnight before treatment with $TPPCS_{2a}$ and light or VEGF/rGel-PCI as discribed above. Three and 24 hrs post light exposure cells were fixed 10 min in 4% paraformaldehyde (PFA), transferred to a humid stain tray, and blocked with 1% BSA in PBS 20 min at room temperature. Cells were then stained for 35 min with Phalloidin-iFluor 594 Reagent from Abcam (#ab176757 1:1000 in PBS) staining actin filaments before they were washed with PBS and stained with primary antibodies αHMGB1 (#ab18256, Abcam 1:1000) or αHSP90 (#4877 CST 1:100) over night at 4°C. Cells were then washed and incubated 30min at room temperature with secondary Goat αrabbit Alexa 488 antibody (#A11034, Life Technologies1:600 in 1%BSA) before they were washed again, incubated 2 min with 0.6 µg/ml Hoechst 3325 (Sigma Aldrich),

washed with PBS prior to dH2O and mounted using ProLong Glass Antifade mountant (Thermo Fisher). The cells were left in the dark over night at room temperature before they were subjected to microscopy using a LSM 880 Airyscan FAST confocal microscope equipped with an Airyscan detector and FAST options, Ar-laser multiline (405/458/488/514/561 and 633 nm and 20x NA 0.8 DIC II (Plan-Apochromat) and 63x NA 1.4 oil DIC III (Plan-Apochromat) objectives (Carl Zeiss AG, Oberkochen, Germany). The Zen blue software (Carl Zeiss AG) was used for image acquisition and processing.

Animals

All animal procedures were performed according to protocols approved by the national animal research authority (Norwegian Food Safety Authority, FOTS ID: 13577) and conducted according to the regulations of the Federation of European Laboratory Animal Science Association (FELASA). All handling of animals were in compliance with EUs Diretive 2010/63/EU on the protection of animals used for scientific purposes. Two different strains of female mice were used in this study; Balb/cJRj and C57BL/6 JRj both obtained from Janiver labs, Saint Berthevin, Pys-de-la-Loire, France and bred at the Department of Comparative Medicine at the Norwegian Radium Hospital, Oslo University Hospital, Norway. The mice were included in the experiments from the age of 7 weeks following at least 1 week of acclimatization. The weight of the animals upon inclusion was 16.1-20.3g for Balb/c and16.4-20.8g for C57BL/6. The mice were maintained under specific pathogen-free conditions in a temperature- and humidity-controlled room. Food and water were supplied ad libitum.

Supplementary figure legends

Fig. S1: Intracellular localization of HSP90 and HMGB1 post VEGF/rGel-PCI. Fluorescence images of CT26 cells showing intracellular localization of A:HSP90 (green) and B: HMGB1(green) 3 and 24 hrs

VEGF121/rGel-PCI with indicated controls. Fluorescence from DAPI (blue), staining the nucleus, and Phalloidin (red), staining the actin filaments, is included for orientation. Yellow color indicates co-localization between green and red. Bar: 10µm. VEGF121/rGel-PCI was performed with the low light dose. All images are representative for 3 independent experiments.

Fig. S2: Relative amount of animals with reduced CT26 volume as compared to day 0. The results are presented as a function of time after treatment initiation. The 4 panels represent increasing doses of αCTLA-4 antibody.

Fig. S3: Relative change in CT26 tumor volume in each animal. The bars represent the relative change in tumor volume for each animal within the different treatment groups at the indicated days after treatment. A-D represents increasing doses of αCTLA-4 antibody.

Fig. S4: CT26 tumor volume distribution at day 4 for all treatment groups. The bars represent the tumor volume for each animal within the different treatment groups at the indicated days after treatment. The animals where scarified when the tumor was larger than 100mm3. CR:Complete response.

Fig. S5: CT26 tumor volume distribution at day 7, 9 and 11 for all treatment groups. The bars represent the tumor volume for each animal within the different treatment groups at the indicated days after treatment. The animals where scarified when the tumor was larger than 100mm3. CR:Complete response.

Fig. S6: Treatment response of CT26 tumors following VEGF/rGel-PCI enhanced αCTLA-4 treatment. A: Kaplan-Meier plots illustrating overall treatment responses following VEGF/rGel-PCI and indicated controls. The panels represent increasing doses of αCTLA-4 antibody. B: Median days to reach endpoint in each treatment group including statistic evaluation of significant differences between the groups. C:

animals in CR at day 9 and 100 following indicated treatment. The table shows the absolute as well as relative number. D shows the growth curves of each individual animal in the indicated treatment groups following VEGF/rGel-PCI enhanced αCTLA-4 (50µgx3) treatment in the presence of an αCD8+ antibody.

Fig. S7: VEGF/rGel-PCI enhanced αCTLA-4 treatment tolerability in the CT26 model. Relative animal weight as compared to day 0 in indicated treatment groups at day 4 (the first 4 bars) and 11 (the last bar). αCTLA-4 antibody was used at a total dose of 150µg (50µgx1 for day for and 50µgx3 for day 11). The bars represent the average of all the animals in each treatment group and error bars show SD. significant difference between the treatment groups is evaluated by ANOVA and the p value is indicated when significant. * indicate significant difference ($p<0.05$) from the other groups.

Fig. S8 Relative amount of animals with reduced MC38 volume as compared to day 0. The results are presented as a function of time after treatment initiation.

Fig. S9: Treatment response of MC38 tumors following VEGF/rGel-PCI enhanced αCTLA-4 treatment. A and B: Relative change in tumor volume from day 0 at indicated days for each individual animal within the treatment groups. A and B represent 0 and 50µgx3 αCTLA-4 antibody. C: Kaplan-Meier plots illustrating overall treatment responses following VEGF/rGel-PCI and indicated controls. The panels represent 0 and 50µgx3 of αCTLA-4 antibody. D: Median days to reach endpoint including statistic evaluation is reported in. E shows animals in CR at day 11 and 100 following treatment in each treatment group. F: shows the growth curves of each individual animal in the indicated treatment groups following VEGF/rGel-PCI enhanced αCTLA-4 (50µgx3) treatment in the presence of an αCD8+ antibody.

Fig. S10: MC38 tumor volume distribution at day 4, 7, 9 and 11 for all treatment groups. The bars represent the tumor volume for each animal within the different treatment groups at the indicated days after treatment. The animals where scarified when the tumor was larger than 100mm3. CR:Complete response.

Fig. S11: VEGF/rGel-PCI enhanced αCTLA-4 treatment tolerability in the MC38 model Relative animal weight as compared to day 0 in indicated treatment groups at day 4 (the first 4 bars) and 11 (the last bar). αCTLA-4 antibody was used at a total dose of 150µg (50µgx1 for day for and 50µgx3 for day 11).The bars represent the average of all the animals in each treatment group and error bars show SD. Significant difference between the groups was evaluated by ANOVA and the p value is indicated when significant. * indicate significant difference ($p<0.05$) from the other groups.

S1

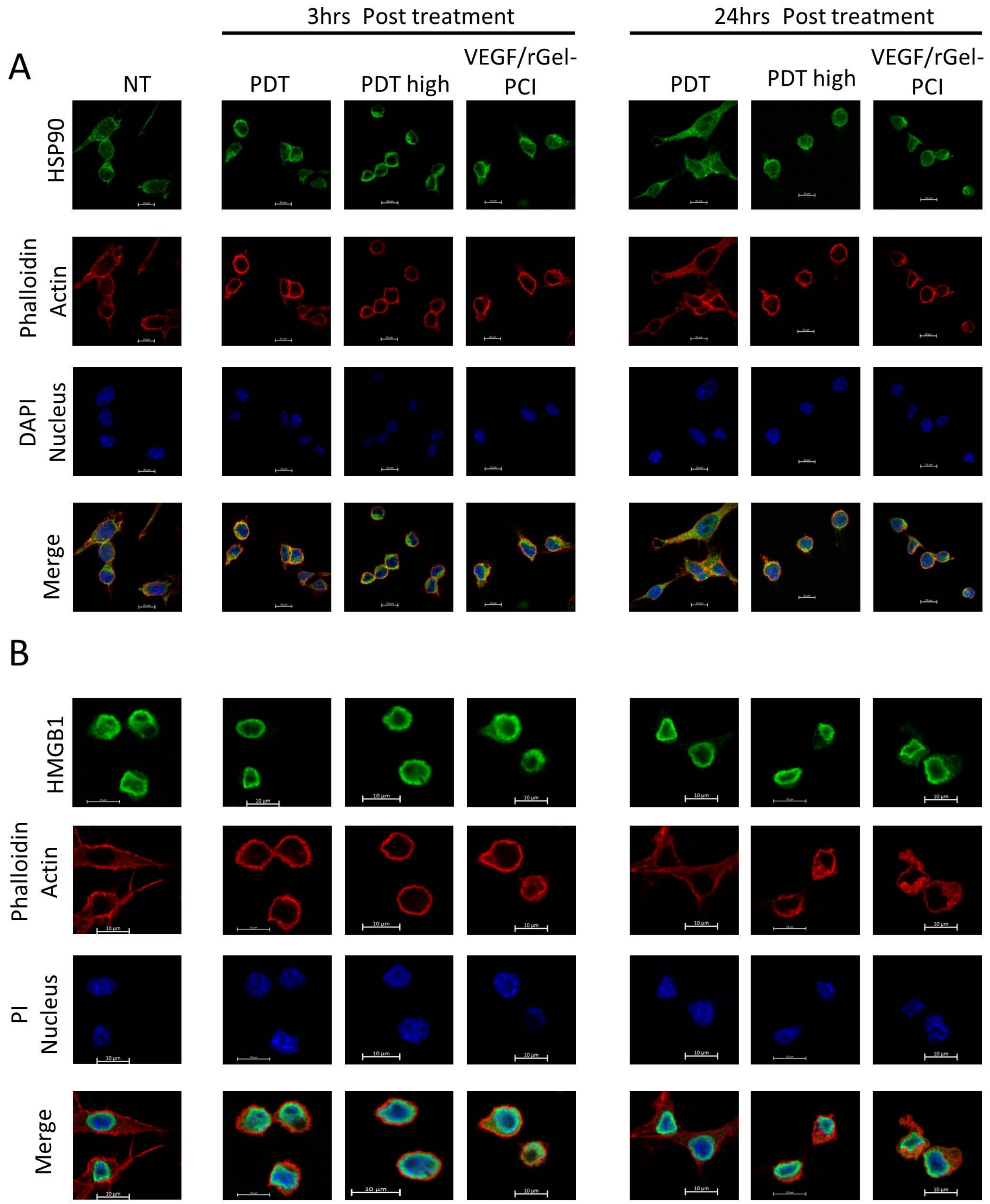

3hrs Post treatment
24hrs Post treatment
A
NT
PDT
PDT high
VEGF/rGel-PCI
PDT
PDT high
VEGF/rGel-PCI
HSP90
Phalloidin Actin
DAPI Nucleus
Merge
B
HMGB1
Phalloidin Actin
PI Nucleus
Merge
10 μm

S2

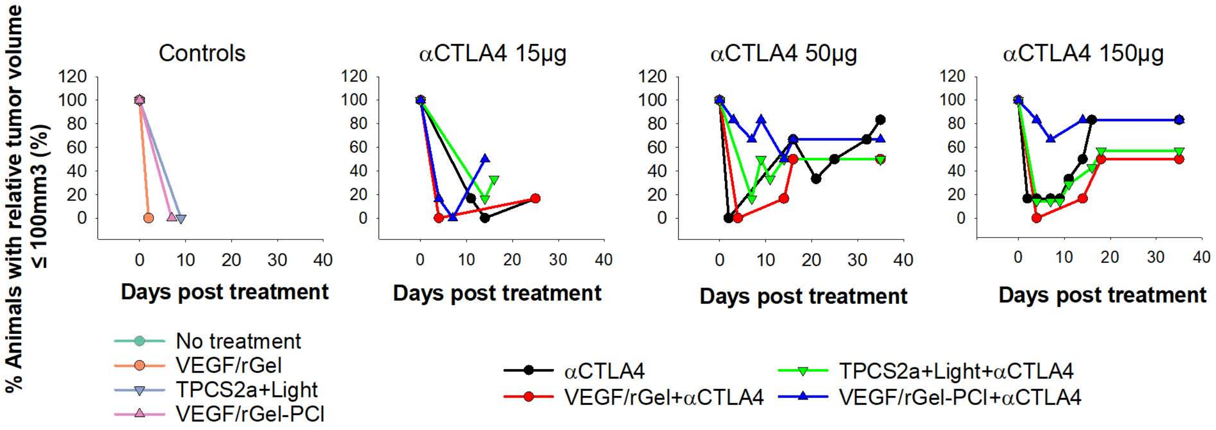
Controls
αCTLA4 15µg
αCTLA4 50µg
αCTLA4 150µg
% Animals with relative tumor volume ≤ 100mm3 (%)
Days post treatment
No treatment
VEGF/rGel
TPCS2a+Light
VEGF/rGel-PCI
αCTLA4
VEGF/rGel+αCTLA4
TPCS2a+Light+αCTLA4
VEGF/rGel-PCI+αCTLA4

S3

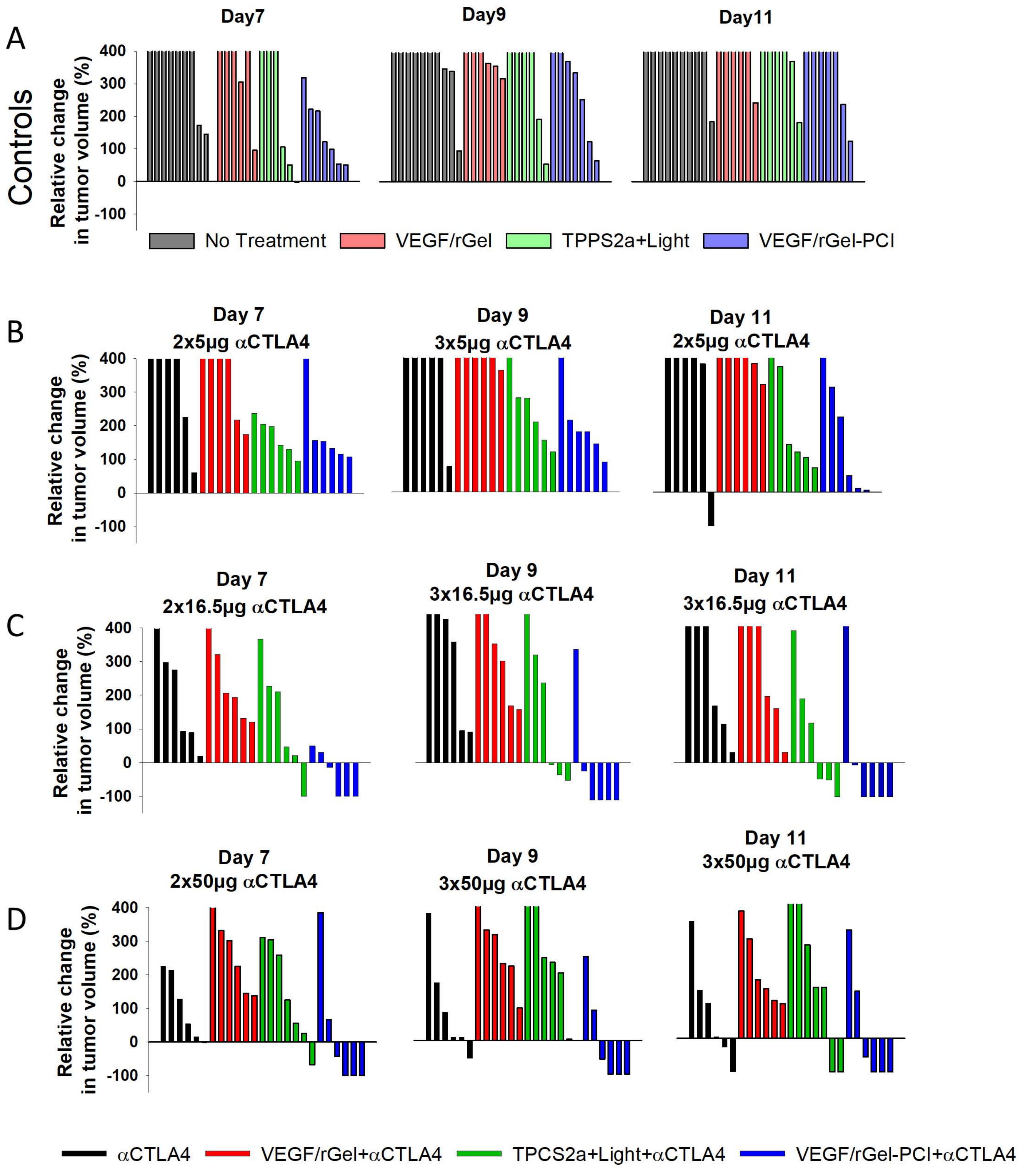
A
Controls
Day7
Day9
Day11
Relative change in tumor volume (%)
400
300
200
100
0
-100
No Treatment
VEGF/rGel
TPPS2a+Light
VEGF/rGel-PCI
B
Day 7
2x5µg αCTLA4
Day 9
3x5µg αCTLA4
Day 11
2x5µg αCTLA4
C
Day 7
2x16.5µg αCTLA4
Day 9
3x16.5µg αCTLA4
Day 11
3x16.5µg αCTLA4
D
Day 7
2x50µg αCTLA4
Day 9
3x50µg αCTLA4
Day 11
3x50µg αCTLA4
αCTLA4
VEGF/rGel+αCTLA4
TPCS2a+Light+αCTLA4
VEGF/rGel-PCI+αCTLA4

S4

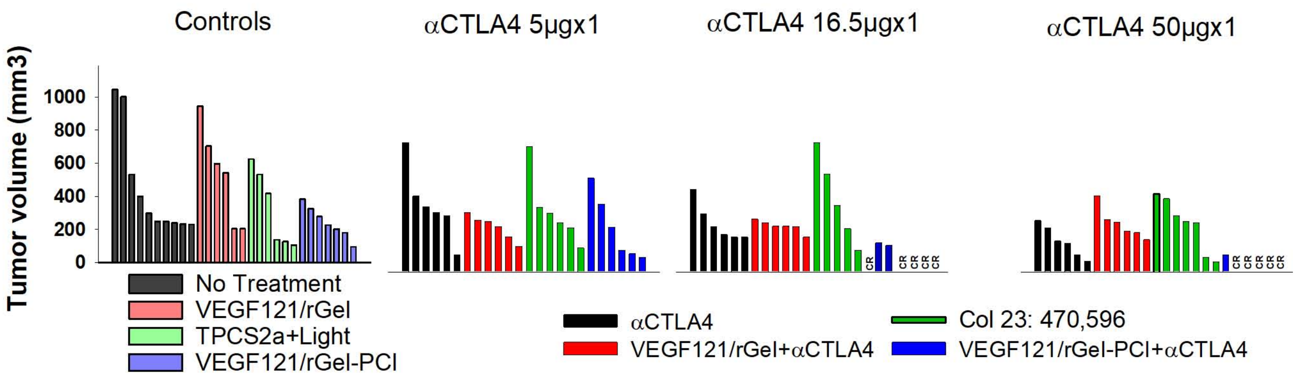

Controls
αCTLA4 5µgx1
αCTLA4 16.5µgx1
αCTLA4 50µgx1
Tumor volume (mm3)
1000
800
600
400
200
0
No Treatment
VEGF121/rGel
TPCS2a+Light
VEGF121/rGel-PCI
αCTLA4
VEGF121/rGel+αCTLA4
Col 23: 470,596
VEGF121/rGel-PCI+αCTLA4
CR

S5

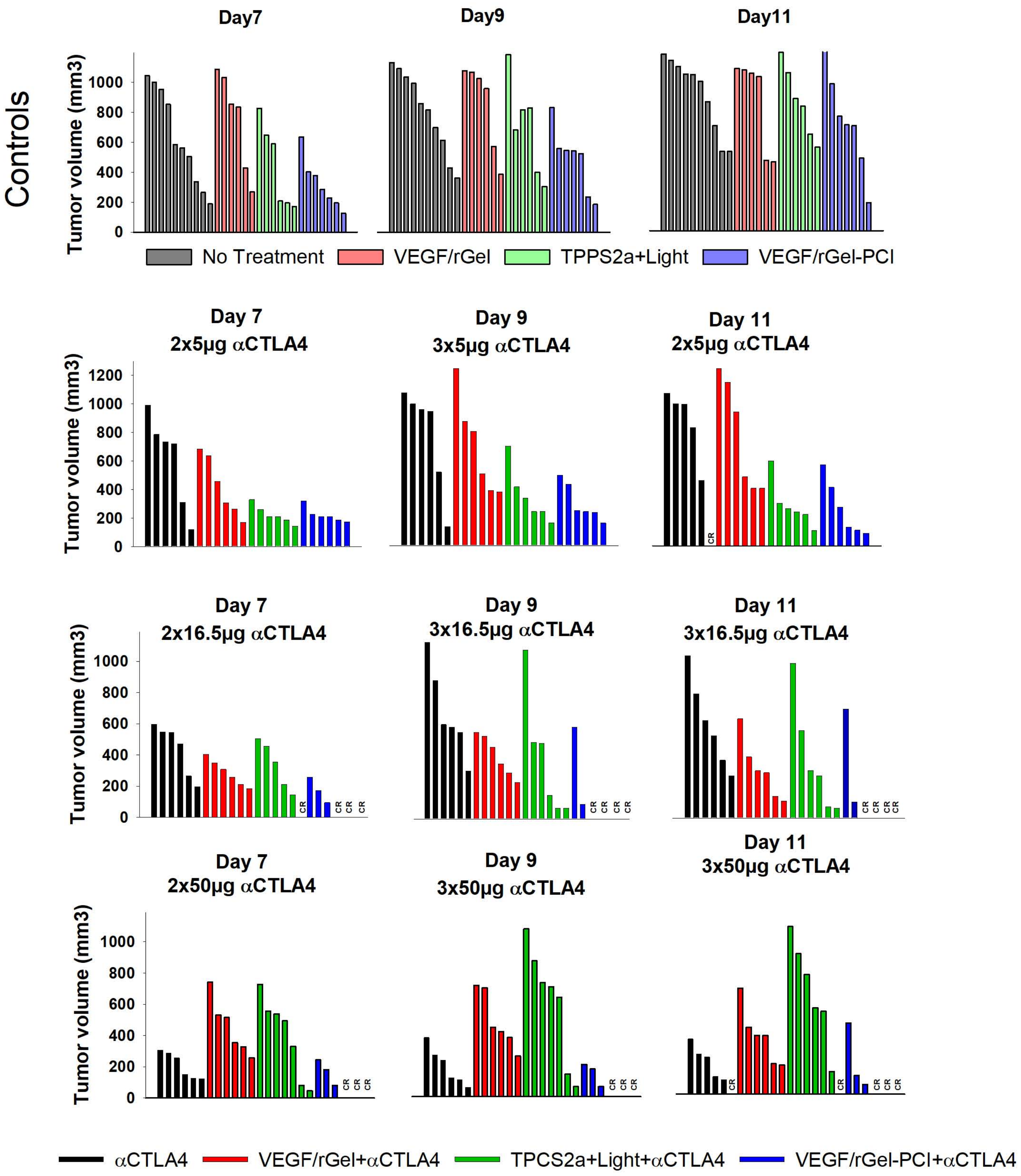
Controls
Day7
Day9
Day11
Tumor volume (mm3)
No Treatment
VEGF/rGel
TPPS2a+Light
VEGF/rGel-PCI
Day 7
2x5μg αCTLA4
Day 9
3x5μg αCTLA4
Day 11
2x5μg αCTLA4
Day 7
2x16.5μg αCTLA4
Day 9
3x16.5μg αCTLA4
Day 11
3x16.5μg αCTLA4
Day 7
2x50μg αCTLA4
Day 9
3x50μg αCTLA4
Day 11
3x50μg αCTLA4
CR
αCTLA4
VEGF/rGel+αCTLA4
TPCS2a+Light+αCTLA4
VEGF/rGel-PCI+αCTLA4

S6

A

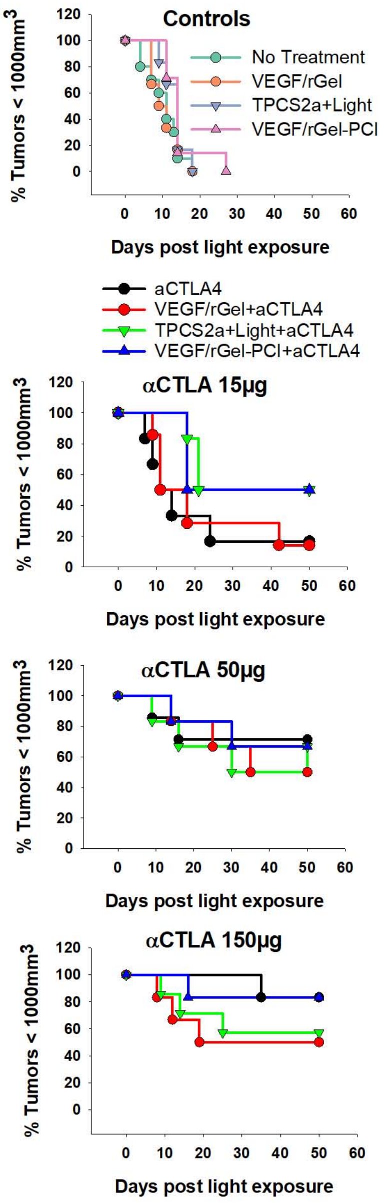

B

CT26: Median days to reach endpoint (1000mm^3)

αCTLA4 dose	0	15µg	50µg	150µg	P value (Log-Rank Test)
Control/αCTLA4	11	11	50	50	<0.001
VEGF/rGel	9	11	35	18	0.009
TPCS2a+Light	13	21	30	50	<0.001
VEGF/rGel-PCI	14	18	50	50	<0.001
P value (Log-Rank Test)	0.385	0.191	0.625	0.403	

αCTLA4 only

Pairwise multiple comparison procedures (Holm-Sidak) Overall significance level = 0.05	
0µg vs 5µgx3	No
0µg vs 16.5µgx3	Yes
0µg vs 50µgx3	Yes
5µx3 vs 16.5µgx3	Yes
5µgx3 vs 50µgx3	Yes
16µgx3 vs 50µgx3	No

VEGF/rGel+αCTLA4

Pairwise multiple comparison procedures (Holm-Sidak) Overall significance level = 0.05	
0µg vs 5µgx3	No
0µg vs 16.5µgx3	Yes
0µg vs 50µgx3	No
5µx3 vs 16.5µgx3	No
5µgx3 vs 50µgx3	No
16µgx3 vs 50µgx3	No

TPCS2a+Light+αCTLA4

Pairwise multiple comparison procedures (Holm-Sidak) Overall significance level = 0.05	
0µg vs 5µgx3	Yes
0µg vs 16.5µgx3	No
0µg vs 50µgx3	Yes
5µx3 vs 16.5µgx3	No
5µgx3 vs 50µgx3	No
16µgx3 vs 50µgx3	No

VEGF/rGel-PCI+αCTLA4

Pairwise multiple comparison procedures (Holm-Sidak) Overall significance level = 0.05	
0µg vs 5µgx3	Yes
0µg vs 16.5µgx3	Yes
0µg vs 50µgx3	Yes
5µx3 vs 16.5µgx3	No
5µgx3 vs 50µgx3	No
16µgx3 vs 50µgx3	No

C

CT26: Animals in CR

	αCTLA4 dose	#animals in CR day 100	% animals CR day 100	# animals in CR day 9	% animals in CR day 9
No Treatment	0	0/10	0	0/10	0
VEGF121/rGel	0	0/6	0	0/6	0
TPCS2a+Light	0	0/6	0	0/6	0
VEGF121/rGel-PCI	0	0/7	0	0/7	0
No Treatment	15µg	1/6	16.6	0/6	0
VEGF121/rGel	15µg	1/6	16.6	0/6	0
TPCS2a+Light	15µg	2/6	33.3	0/6	0
VEGF121/rGel-PCI	15µg	3/6	50	0/6	0
No Treatment	50µg	4/6	66.7	0/6	0
VEGF121/rGel	50µg	3/6	50	0/6	0
TPCS2a+Light	50µg	3/6	50	0/6	0
VEGF121/rGel-PCI	50µg	4/6	66.7	4/6	66.7
No Treatment	150µg	5/6	83.3	0/6	0
VEGF121/rGel	150µg	3/6	50	0/6	0
TPCS2a+Light	150µg	4/6	66.7	0/6	0
VEGF121/rGel-PCI	150µg	5/6	83.3	3/6	50

D

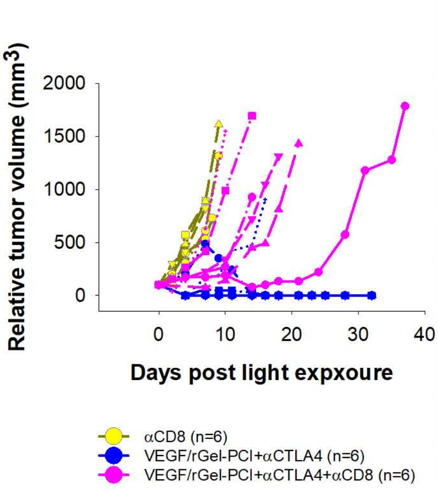

S7

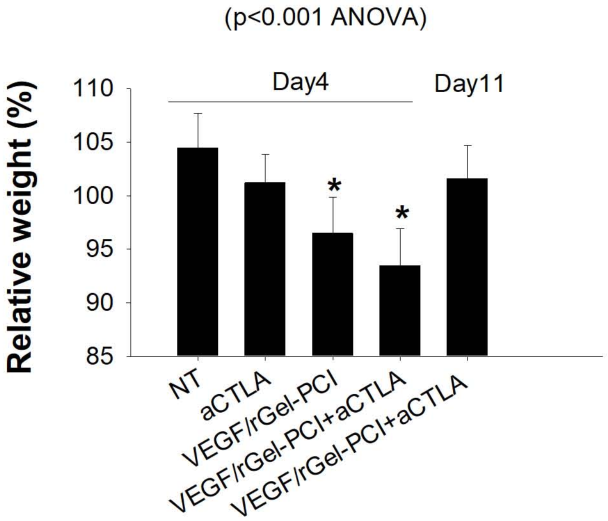

(p<0.001 ANOVA)
Day4
Day11
Relative weight (%)
110
105
100
95
90
85
*
*
NT
aCTLA
VEGF/rGel-PCI
VEGF/rGel-PCI+aCTLA
VEGF/rGel-PCI+aCTLA

S8

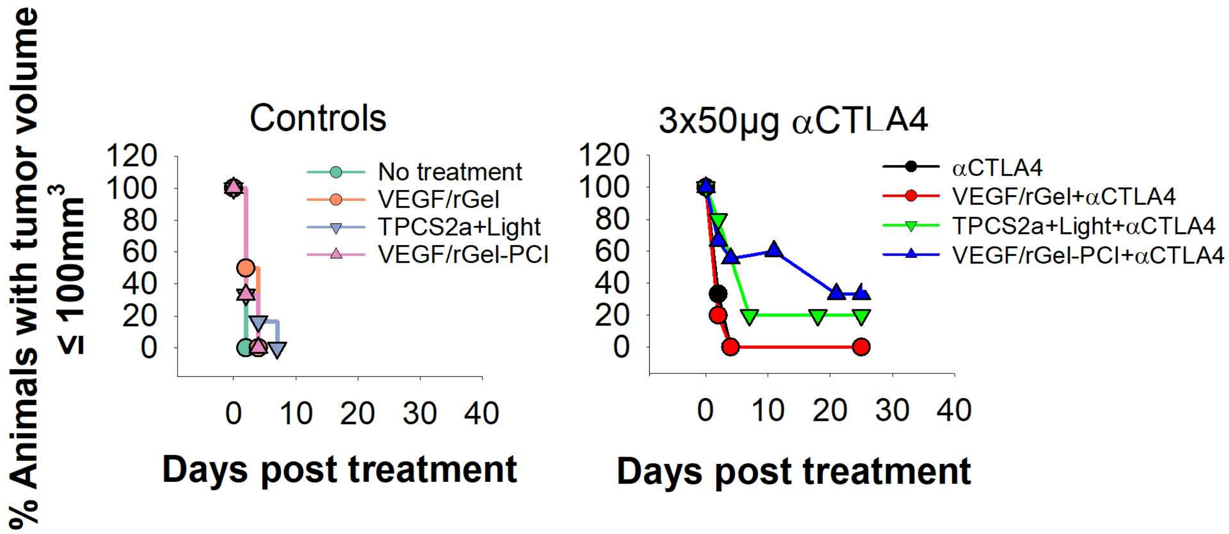

% Animals with tumor volume ≤ 100mm^3
Controls
120
100
80
60
40
20
0
No treatment
VEGF/rGel
TPCS2a+Light
VEGF/rGel-PCI
0
10
20
30
40
Days post treatment
3x50µg αCTLA4
αCTLA4
VEGF/rGel+αCTLA4
TPCS2a+Light+αCTLA4
VEGF/rGel-PCI+αCTLA4
Days post treatment

S9

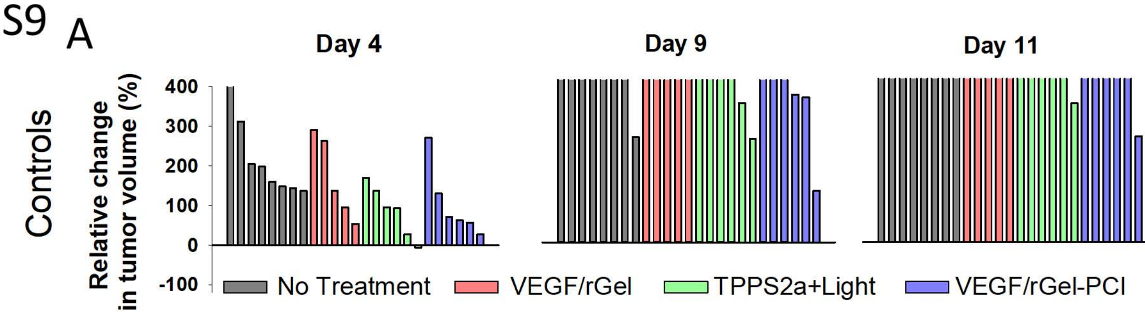

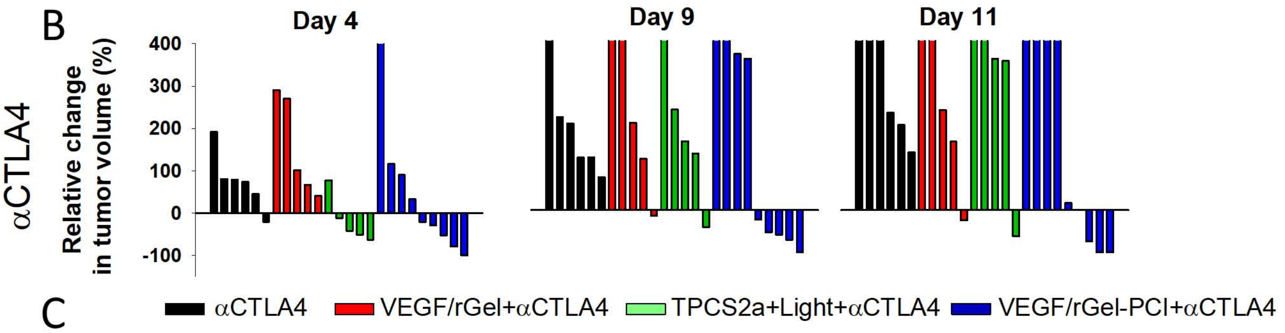

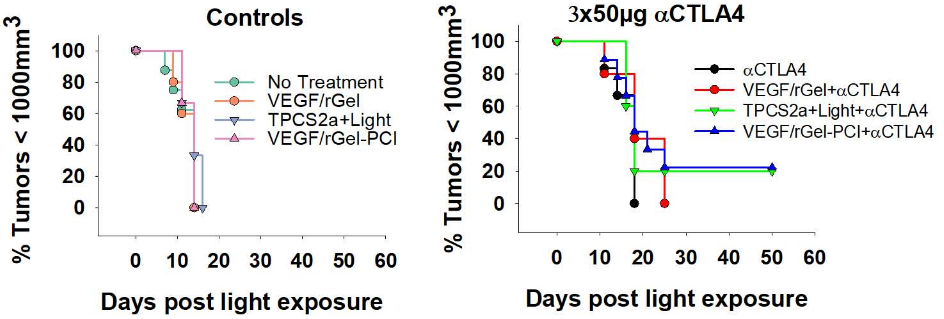

D

MC38: Median days to reach endpoint (1000mm^3)

αCTLA4 dose	0	150μg	P value (Log-Rank Test)
Control/αCTLA4	13	17	0.011
VEGF/rGel	12	18	0.025
TPCS2a+Light	14	17	0.016
VEGF/rGel-PCI	13	17	0.006
P value (Log-Rank Test)	0.139	0.594	

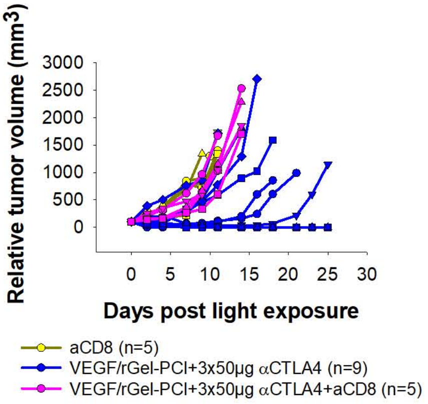

E

MC38: Animals in CR

	αCTLA4 dose	#animals in CR day 100	% animals CR day 100	# animals in CR day 11	% animals in CR day 11
No Treatment	0	0/8	0	0/8	0
VEGF121/rGel	0	0/5	0	0/5	0
TPCS2a+Light	0	0/6	0	0/6	0
VEGF121/rGel-PCI	0	0/6	0	0/6	0
No Treatment	150μg	0/6	0	0/6	0
VEGF121/rGel	150μg	0/5	0	0/5	0
TPCS2a+Light	150μg	1/5	20	0/5	0
VEGF121/rGel-PCI	150μg	2/9	22	2/9	22

S10

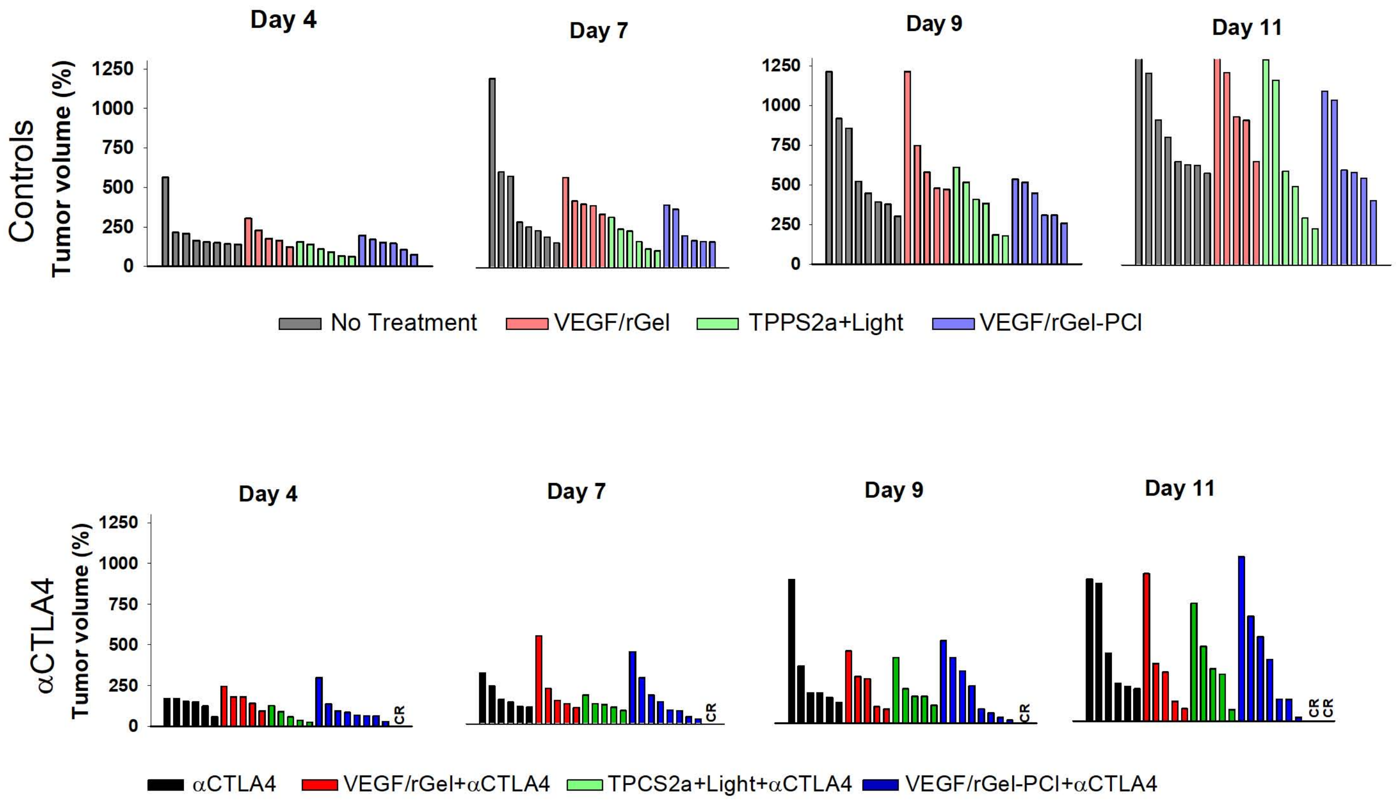
Day 4
Day 7
Day 9
Day 11
Controls
Tumor volume (%)
1250
1000
750
500
250
0
No Treatment
VEGF/rGel
TPPS2a+Light
VEGF/rGel-PCI
αCTLA4
CR
αCTLA4
VEGF/rGel+αCTLA4
TPCS2a+Light+αCTLA4
VEGF/rGel-PCI+αCTLA4

S11

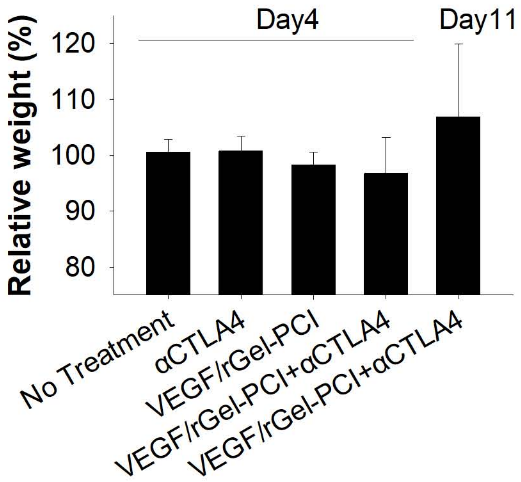
Relative weight (%)
120
110
100
90
80
Day4
Day11
No Treatment
αCTLA4
VEGF/rGel-PCI
VEGF/rGel-PCI+αCTLA4
VEGF/rGel-PCI+αCTLA4
